# Local field potentials and single unit dynamics in motor cortex of unconstrained macaques during different behavioral states

**DOI:** 10.3389/fnins.2023.1273627

**Published:** 2023-11-23

**Authors:** Richy Yun, Irene Rembado, Steve I. Perlmutter, Rajesh P. N. Rao, Eberhard E. Fetz

**Affiliations:** ^1^Department of Bioengineering, University of Washington, Seattle, WA, United States; ^2^Center for Neurotechnology, University of Washington, Seattle, WA, United States; ^3^Washington National Primate Research Center, University of Washington, Seattle, WA, United States; ^4^Department of Physiology and Biophysics, University of Washington, Seattle, WA, United States; ^5^Allen School of Computer Science and Engineering, University of Washington, Seattle, WA, United States

**Keywords:** brain states, non-human primates, LFP, cross-frequency phase-amplitude coupling, extracellular single units, spike-LFP synchrony

## Abstract

Different sleep stages have been shown to be vital for a variety of brain functions, including learning, memory, and skill consolidation. However, our understanding of neural dynamics during sleep and the role of prominent LFP frequency bands remain incomplete. To elucidate such dynamics and differences between behavioral states we collected multichannel LFP and spike data in primary motor cortex of unconstrained macaques for up to 24 h using a head-fixed brain-computer interface (Neurochip3). Each 8-s bin of time was classified into awake-moving (Move), awake-resting (Rest), REM sleep (REM), or non-REM sleep (NREM) by using dimensionality reduction and clustering on the average spectral density and the acceleration of the head. LFP power showed high delta during NREM, high theta during REM, and high beta when the animal was awake. Cross-frequency phase-amplitude coupling typically showed higher coupling during NREM between all pairs of frequency bands. Two notable exceptions were high delta-high gamma and theta-high gamma coupling during Move, and high theta-beta coupling during REM. Single units showed decreased firing rate during NREM, though with increased short ISIs compared to other states. Spike-LFP synchrony showed high delta synchrony during Move, and higher coupling with all other frequency bands during NREM. These results altogether reveal potential roles and functions of different LFP bands that have previously been unexplored.

## Introduction

Previous studies have furthered our understanding of different sleep stages by exploring the dynamics of local field potential (LFP) frequency band power and single-unit spiking characteristics in both the cortex and various deep brain structures (Brown et al., 2012; Rasch and Born, 2013). Slow waves and delta frequency band are typically present across the brain during NREM sleep, and high theta power is present during REM sleep (Rechtschaffen and Kales, 1968; Silber et al., 2007). Single units show changes in firing rate as well as firing patterns depending on the sleep stage due to changes in excitability (Tononi and Cirelli, 2014; Xu et al., 2019; Arbune et al., 2020). In particular, the motor cortex shows increased excitability and sequential firing of neurons during REM sleep which may play a role in memory consolidation (Hess et al., 1987; Xu et al., 2019), and displays reactivations of relevant cortical circuit patterns to solidify motor learning during NREM sleep (Ramanathan et al., 2015; Xu et al., 2019; Rubin et al., 2022). Several other features of cortical activity, such as k-complexes and sleep spindles during NREM sleep, have also been shown to be associated with different stages of sleep (Huber et al., 2004; Ulrich, 2016; Fernandez and Lüthi, 2020). However, the neural dynamics underlying sleep and the functional correlates of the prominent LFP bands remain unclear.

Various measures of local field potential (LFP) coupling and spike-LFP synchrony have been commonly used to elucidate how brain networks communicate for learning, memory, and various cortical functions (Einevoll et al., 2013; Khanna and Carmena, 2017; Telenczuk et al., 2017). Cross-frequency phase-amplitude coupling of different LFP frequency bands, thought to reflect communication between brain circuitry, has been shown to be modulated by task performance, cognitive engagement, and memory formation in various brain regions (Jensen and Colgin, 2007; Canolty and Knight, 2010). Single units are strongly synchronized to specific phases of LFP frequency bands, notably to beta and gamma cycles in the motor cortex, suggesting beta to be a resting rhythm during movement execution and gamma to reflect local population activity (Murthy and Fetz, 1996a,b; Engel and Fries, 2010; Buzsáki et al., 2012; Buzsáki and Wang, 2012). LFP coupling in the motor cortex during sleep is not as common, typically used to investigate Parkinson’s disease (Sanabria et al., 2017; Devergnas et al., 2019). LFP and unit analyses have given us significant insight into neural signaling and the role of both spikes and LFPs in brain function, but there has yet to be a comprehensive investigation of how cross-frequency coupling and spike-LFP synchrony for specific cortical sites are modulated by behavioral states during sleep and wakefulness.

This study analyzes LFPs, single units, and their relationship in the macaque motor cortex during different behavioral states to clarify the mechanisms underlying neural dynamics of these states as well as to elucidate the roles of various LFP frequency bands in cortical communication. We first used the power spectral density of LFPs to distinguish between four major behavioral states shown to be relevant to plasticity and learning in the motor cortex: (1) awake and moving (Move), (2) awake and at rest (Rest), (3) rapid-eye movement (REM) sleep, and (4) non-REM (NREM) sleep. We tracked single-unit activity concurrently with LFP recordings and focused on 6 different frequency bands commonly delineated in the cortex: (1) delta (0.5–4 Hz), (2) theta (4–8 Hz), (3) alpha (8–12 Hz), (4) beta (15–30 Hz), (5) low gamma (30–70 Hz), and (6) high gamma (70–120 Hz). Finally, we assessed state-dependent changes in cross-frequency coupling between pairs of the frequency bands as well as spike-LFP synchrony in each frequency band. The results show that the relationships between LFP bands depend on the animal’s behavioral state and that spike-LFP synchrony may provide insight into the underlying mechanisms.

## Materials and methods

### Experimental design

#### Implants and surgical procedures

The experiments were conducted on two male *Macaca nemestrina* monkeys (Monkeys J and K). Surgeries were performed under isoflurane anesthesia and aseptic conditions to implant multi-electrode Utah arrays. All procedures conformed to the National Institutes of Health *Guide for the Care and Use of Laboratory Animals* and were approved by the University of Washington Institutional Animal Care and Use Committee.

Implantation of the Utah array was guided by stereotaxic coordinates. A 1.5 cm wide square craniotomy was performed over the hand region of the primary motor cortex, as determined by stereotactic coordinates, to expose the dura. Three sides of the exposed dura were cut to expose the cortex; a Utah array was then implanted and the dura was sutured over the array. Two reference wires were inserted below the dura and two were inserted between the dura and the skull. The bone flap from the craniotomy was replaced and held in place by a titanium strap screwed onto the skull with 2.5 mm × 6 mm titanium skull screws. A second smaller titanium strap was fastened to the skull to secure the wire bundle. The connector pedestal for the array was attached to the skull with eight titanium skull screws and the incision closed around the pedestal base. Additional skull screws were placed around the base of the connector pedestal and a thin coat of dental acrylic (methyl methacrylate) was applied to the skull between the screws and the connector base for additional stability.

To facilitate long-term recordings while the animal is freely behaving in its cage, the animals also received a “halo” implant to house the recording device. The implant was made with 3/8″ aluminum forming an egg-shaped oval 17 cm long and 15.3 cm wide. Four titanium straps were affixed to the skull by titanium bone screws. Two were implanted bilaterally over the occipital ridge, and two were placed temporally bilaterally. After the plates integrated with the skull for 6 weeks, an aluminum halo was mounted on four pins seated in each plate. A titanium can with a plexiglass top to house the Neurochip3 (Shupe et al., 2021, see the *Electrophysiology* section) was attached to the halo during all Neurochip3 recording sessions.

Monkey J additionally received electrooculogram (EOG) electrodes on the lateral wall and superior margin of both orbits. EOG electrodes consisted of a titanium washer (#4, 0.25” OD, 0.032″ thick) with a 0.016″ hole drilled into it and a 34-gauge silver-plated copper microwire with silicone shielding (Cooner Wire, AS155-34) threaded through the hole and soldered to the washer. An incision was made to the dorsal and lateral margins of both orbits to expose the skull. A small hole was drilled in each incision, a titanium skull screw (2 mm × 6 mm) was used to hold down the EOG electrodes, and the wires of the electrodes were tunneled beneath the tissue along the skull. Another incision was made along the top of the skull to secure a pedestal with 8 skull screws and allow access to the electrode wires. The inside of the pedestal was filled with surgical silicone adhesive (Kwik-Sil, World Precision Instruments) to prevent infection. Connectors were fastened to the wires postoperatively.

After each surgery animals received postoperative courses of analgesics and antibiotics. Animals did not show signs of discomfort or pain related to any implanted devices after recovery, and all exposed implants were regularly disinfected biweekly with chlorohexidine and treated with antibiotics to prevent infection.

#### Electrophysiology

**Figure 1:**
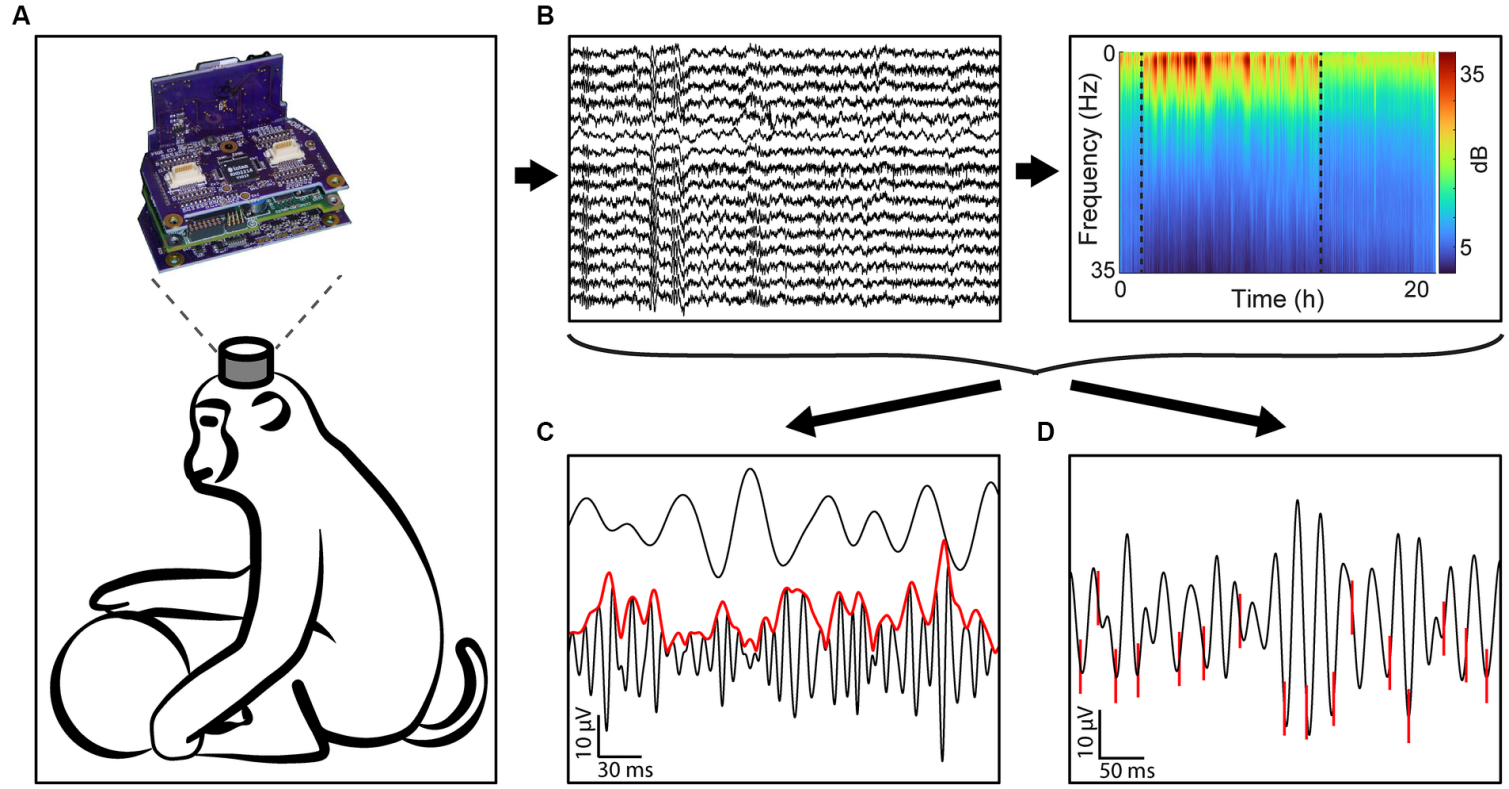
Experimental design. **(A)** All data was gathered using the Neurochip3 on freely behaving macaques for up to 24 h. **(B)** We collected 16 channels of data at 20 kHz and obtained the spectral density of every 8-s bin to classify behavioral states. **(C)** We then calculated the cross-frequency phase-amplitude coupling of every frequency band pair. The example shows the instantaneous amplitude (red) of high gamma filtered LFP (black, bottom) increasing at the trough of beta (black, top). **(D)** We additionally sorted single units and found the synchrony of spikes to LFP bands. The example shows spikes (red) synchronized with the trough of beta filtered LFP.

All data was collected with the head-mounted Neurochip3 (Shupe et al., 2021) while the monkeys were freely behaving in their home cage (Figure 1A). The Neurochip3 is a battery powered bidirectional brain-computer interface capable of saving data to an SD card, allowing for wireless recording and stimulation for up to 24 h. Sixteen channels of the Utah array, or 14 channels of the array and 2 channels of the EOG, were recorded at 20 kHz sampling rate with a bandwidth of 0.1 Hz to 5 kHz (Figure 1B). The cortical channels were chosen each day with a preliminary recording to capture the largest single units present in the array. Most experiments recorded a different set of channels, depending on the best spike recordings.

The Neurochip3 also has an onboard 3-axis accelerometer that was simultaneously recorded at 100 samples per second. Recording sessions lasted between 19 and 24 h. The lights in the animal rooms were off during the night for the 12 h between 6 pm and 6 am. We collected data from a total of 16 sessions from Monkey K and 10 sessions from Monkey J.

Three sessions from Monkey J were recorded immediately following sedation via intramuscular ketamine injection to facilitate mounting the Neurochip3. We removed the first 3 h of recordings following recovery from these sessions to ensure the data was not affected by the sedation. No results were different when removing these sessions, so they were included to be comprehensive.

### Data analysis

#### Classifying sleep states

Although there have been various approaches automating sleep state classification by taking advantage of the sequential nature of the states as suggested by the American Academy of Sleep Medicine (AASM) classification (Silber et al., 2007; Supratak et al., 2017; Craik et al., 2019), studies have shown that including information from neighboring epochs does not necessarily improve classification (Tsinalis et al., 2016; Sekkal et al., 2022). Most deep learning methods also rely on supervised learning, but, due to the high inter-scorer variability in manual classification that these models rely on (Himanen and Hasan, 2000; Younes et al., 2016), we chose an unsupervised method instead. As a result, we used dimensionality reduction for feature extraction and subsequent clustering. Autoencoders were chosen as the method for dimensionality reduction which, due to their nonlinearity, are potentially able to extract more salient features compared to linear methods. Denoising autoencoders are often used for feature extraction to ensure the network does not learn to replicate consistent noise, but our input data inherently contained random noise due to the recording device being mounted on a freely behaving animal as well as the short time window of 8 s for our power spectral density calculations. As a result, we used a stacked sparse autoencoder for dimensionality reduction.

All analyses were performed using custom MATLAB (MathWorks) and Python code. The power spectral density (PSD) of cortical local field potentials (LFPs) as well as the onboard accelerometer data from the Neurochip3 were used to classify different sleep states (Figure 1B). Data was down sampled to 1 kHz before performing Welch’s PSD estimate between 0 and 50 Hz for each 8 s time-bin. We then converted the PSD into power and found the average power across all channels for each time-bin. The average power was further normalized by subtracting the minimum value and dividing by the integral to ensure that relative power at different frequencies played a larger role than the absolute power.

We then used the normalized average power as inputs to train a stacked sparse autoencoder for dimensionality reduction, similar to the architecture described in Tsinalis et al. (2016). The encoder was composed of 3 layers of 256, 128, and 64 units each, with batch normalization and ReLU activation function. The final hidden layer containing the reduced representation of the data had 32 nodes. The autoencoder was trained with minibatch sizes of 64 for 300 epochs. The loss was calculated with mean squared error with L1 regularization (regularization weight $\lambda =1e^{-5}$ ) using the Adam optimizer (learning rate $\alpha =1e^{-3}$ , decay rate for first moment $\beta_{1}=0.9$ , decay rate for second moment $\beta_{2}=0.999$ , constant $\varepsilon =1e^{-8}$ ). The autoencoder was implemented in Python using the PyTorch package (Paszke et al., 2019).

Accelerometer data was included by performing the root sum of squares across all three axes. We then found the variance of the values within each time bin and applied a logarithmic scale to better compress the data. Finally, the standard deviation was normalized to that of the encoded dimension with the largest variance. The processed accelerometer data was included as an additional dimension in the lower dimensional representation (i.e., as the 33rd dimension).

To classify the data, we used k-means clustering with an assumption of 4 centroids. Data points of the lower dimensional representation within the 90th percentile of pairwise Euclidean distance were initially used for finding the centroids of clusters to avoid the influence of outliers. Each data point was assigned to the centroid with the shortest Euclidean distance.

After clustering, each group of records was assigned to one of four states – (1) awake and moving (Move), (2) awake and at rest (Rest), (3) rapid-eye movement (REM) sleep, (4) non-REM (NREM) sleep – by assessing the average accelerometer value and average normalized PSD for each cluster. First, the cluster with highest average acceleration was assigned to be Move, then the cluster with highest average delta power (0.5–4 Hz) was assigned be NREM, then the cluster with higher average beta power (15–30 Hz) was assigned to be Rest, and the remaining cluster was assigned to be REM.

To include a temporal aspect and smooth any outliers we performed a majority filter on the classification. For each time-bin, the majority state across 2 time-bins before to 2 time-bins after (±16 s) was considered to be the current state. Ties were resolved by keeping the original classification, or by random choice if the original classification was not part of the tie.

#### Validation of classification

Two EOG electrodes (one dorsal and one lateral) were simultaneously recorded from with the Neurochip3 in experiments with Monkey J. EOG signals were extracted by subtracting the lateral electrode signal from the dorsal electrode signal and then applying a band-pass filter between 1 and 20 Hz with a zero-phase second-order Butterworth filter. We performed in-booth experiments with flashes of lights guiding the monkey’s gaze to ensure we were properly capturing eye movements.

As the Neurochip3 is mounted to the monkeys’ heads, we performed overnight recordings with the Microsoft Kinect in conjunction to ensure there were no large discrepancies between the on-board accelerometer and whole-body movements (Libey and Fetz, 2017). With the Kinect movements were calculated as the absolute difference between each frame of the infrared depth-finding camera and the previous frame. We captured frames as quickly as possible with the processing overhead, around 30 frames per second.

Further validation was performed with k-fold cross-validation with *k* = 20. Each dataset was split into 20 random groups; classification was performed on 19 of the 20 groups, and the final group was classified using the centroids from the classification k-means. The training error was calculated as the difference in classification within the 19 groups used for training the classification, and the test error as the difference in classification within the final group used to test the classification. Cross-validation was bootstrapped 50 times for each session to minimize variability that can potentially be introduced by the random sampling.

#### Coherence

Magnitude-squared coherence was used to calculate synchrony within the same frequencies:


(1) $$C_{xy}=\frac{{\left|P_{xy}\left(f\right)\right|}^{2}}{P_{xx}\left(f\right)P_{yy}\left(f\right)}$$

where $C_{xy}$ is the coherence between x and y , $P_{xy}\left(f\right)$ is the cross-spectral density between x and y , and $P_{xx}\left(f\right)$ and $P_{yy}\left(f\right)$ are the spectral densities of x and y respectively. Coherence was calculated between all combinations of channel pairs every 0.1 Hz.

#### Cross-frequency phase-amplitude coupling

To calculate cross-frequency phase-amplitude coupling we used mean vector length (MVL) (Canolty et al., 2006; Figure 1C). The LFP was first filtered into 2 frequency bands of interest within the 6 frequency bands of interest – (1) delta (0.5–4 Hz), (2) theta (4–8 Hz), (3) alpha (8–12 Hz), (4) beta (15–30 Hz), (5) low gamma (30–70 Hz), and (6) high gamma (70–120 Hz) – using a zero-phase second-order Butterworth filter. We then calculated the analytic signal, H , of each band using the Hilbert transform:


(2) $$H\left(x\left(t\right)\right)=\frac{1}{\pi }P\overset{+\infty }{\underset{-\infty }{\int }}\frac{x\left(\tau \right)}{t-\tau }d\tau$$

where P is Cauchy principal value. The phase of the complex valued analytic signal is the instantaneous phase at time t , and the magnitude of the analytic signal is the instantaneous amplitude. The mean vector of the two considered frequency bands was then calculated by:


(3) $$MVL=\left|\frac{1}{n}\underset{n}{\sum }r\;\exp\;\left(i\phi \right)\right|$$

where r is the instantaneous magnitude of the higher frequency band and ϕ is the instantaneous phase of the lower frequency band. ϕ ranges from 0 to 2 π radians where 0 is the peak and π the trough of oscillations. This calculation creates a vector for each sample in time with the phase and amplitude of the lower and higher frequency bands, respectively. The magnitude of the average across all these vectors, or MVL, measures the strength of synchrony – zero indicates a uniform distribution in which the vectors “cancel” each other, and higher values indicate the degree of synchrony.

Mean vector length is highly affected by the amplitude and does not have a normalized upper limit, which makes interpretation of individual values and comparisons of MVL measurements across different time points difficult. Thus, we additionally calculated the maximum possible MVL for each state. Instead of using the phase and amplitude that occurs at the same time sample in Equation 2, we paired the highest amplitudes with the most commonly occurring phases within each behavioral state. Thus, the largest vectors were all in similar directions providing the maximum possible length of the mean vector. We then normalized the MVL by dividing it by the maximum possible MVL to assess differences in synchrony between behavioral states.

#### Spike sorting

Spikes were sorted offline using two-window discrimination. The cortical recording was bandpass filtered between 1,000 and 2000 Hz with a first-order Butterworth filter. Then a negative threshold and two time-delayed windows were manually chosen to capture the trough and the peak of the spike waveform. All traces crossing the threshold and passing through the two windows were denoted as spikes.

As the fidelity of spikes may change over such a long period of recording, we additionally compared the shapes of the first 1,000 detected spikes with the last 1,000 detected spikes using the coefficient of determination (CoD):


(4) $$\mathrm{CoD}=1-\frac{\sum {\left(s_{\mathrm{first}}\left(t\right)-s_{\mathrm{last}}\left(t\right)\right)}^{2}}{\sum {\left(s_{\mathrm{first}}\left(t\right)-\bar{s_{\mathrm{first}}}\right)}^{2}}$$

where $s_{\mathrm{first}}$ is a waveform of one of the first spikes, $s_{\mathrm{last}}$ is a waveform of one of the last spikes, and $\bar{s_{\mathrm{first}}}$ is the average of the waveform.

We performed pairwise CoD on all first and last 1,000 instances of each spike and compared them to the pairwise CoD between the first 1,000 instances of the spike and the last 1,000 instances of a different, randomly chosen spike. If the distribution of the CoD comparing the same spike was significantly higher than the distribution between the spike and another spike it was considered to be consistent overnight. Additionally, to ensure we were not capturing multiple units with a similar waveform, we manually assessed the autocorrelograms for each spike to ensure the presence of a refractory period and proper distribution of inter-spike intervals.

Additionally, spikes were designated to originate from regular firing or fast firing neurons. Although single unit classification was traditionally performed with the spike width (McCormick et al., 1985), subsequent studies have suggested that spike width, at least when used alone, may not be the most indicative of the type of neuron (Jung et al., 1998; Vigneswaran et al., 2011; Insel and Barnes, 2015). Assessment of various features of spikes showed that the peak of the inter-spike interval (ISI) distribution to be the most distinguishable feature within our dataset (Supplementary Figure S1). If the peak occurred before 10 ms the single unit was designated to be fast spiking (FS), otherwise it was designated to be regular spiking (RS) (84 FS units, 109 RS units). Although we determined the best method to distinguish between RS and FS units, this analysis is presented as a first step in assessing cell type; intracellular recording or other imaging techniques are necessary to establish the differences and determine subclassifications with more confidence (Barthó et al., 2004).

#### Phase-locking value

We calculated the phase-locking value (PLV) to assess the strength of synchronization of spike timing to phases of oscillations in specific frequency bands from LFPs recorded on the same channel (Figure 1D). First, the LFP was filtered into a frequency band of interest. The instantaneous phase of the lower frequency band was calculated through the Hilbert transform as described above (*Cross-frequency phase-amplitude coupling*, Equation 2). We then calculated the PLV:


(5) $$PLV=\frac{1}{n}\left|\underset{n}{\sum }\exp\;\left(i\phi \right)\right|$$

where ϕ is the instantaneous phase at spike times. The PLV effectively converts each phase into a unitary vector and finds the average of all the vectors. The magnitude of the resulting vector determines the synchrony of the phases. A value of zero, similar to MVL, indicates a uniform distribution, or no synchrony. A value of one indicates that all phases are equal, or perfect synchrony.

The average phase of spike timing was found using the circular mean:


(6) $$\mathrm{average}\;\mathrm{phase}=\arg\left(\underset{n}{\sum }\exp\;\left(i\phi \right)\right)$$

where arg is the argument of a complex number, or the angle between the positive real axis and the complex number in the complex plane.

Although measures of spike-field coherence including the PLV can be affected by the number of spikes used in the calculation (Maris et al., 2007), bootstrapped analysis shows that the bias introduced by the number of spikes becomes inconsequential after 10,000 spikes (Supplementary Figures S2B,C). PLV was calculated on spikes of an individual single unit during a single state for each session; >98% of the instances had more than 10,000 spikes (Supplementary Figure S2A).

#### Statistical analysis

We used Friedman’s test to compare values between the four states due to the non-parametric nature of the distribution and because we were sampling the same spikes and LFPs across each state. Tukey’s honest significance test was used *post hoc* to determine significant pairwise differences. Statistical significance was determined to be when the value of *p* was less than 0.05 for each corresponding statistical test.

## Results

### Classification of sleep states

Sleep states were classified using the power spectral density of local field potentials (LFPs) of successive 8-s time-bins as the input to train an autoencoder. We then extracted the values from the hidden units as the low-dimensional representation of the data. Supplementary Figure S3A shows examples of the original spectra and reconstructed outputs after training the autoencoder. The fluctuations in the spectra have been smoothed out but the salient features, such as peaks at alpha or beta, were maintained. Supplementary Figure S3B shows the low-dimensional representation visualized via t-distributed stochastic neighbor embedding (t-SNE) (van der Maaten and Hinton, 2008) after full classification. There was some overlap between the states in the representation, which is to be expected as brain states during sleep are on a continuous spectrum. Finally, we wanted to gain insight into which feature each of the hidden units of the autoencoder represent. Supplementary Figure S3C shows the average of 100 spectra that gave the largest values in each hidden unit and Supplementary Figure S3D shows the average of 100 spectra that gave the smallest values in each hidden unit subtracted from spectra in Supplementary Figure S3C. This analysis can be interpreted as the features within the spectra that each hidden unit encodes, and clear peaks in each frequency band can be observed.

We then applied k-means clustering on the lower dimensional representation as well as the accelerometer data that was collected concurrently through the Neurochip3. We assumed 4 clusters – awake and moving (Move), awake and at rest (Rest), REM sleep (REM) and non-REM sleep (NREM). Each cluster was assigned to the state depending on the average accelerometer value and features in the average power spectral density (PSD). Although we have a physiological basis as to why we chose 4 clusters, we wanted to ensure that 4 clusters were a reasonable number for our dataset. To that end, we tracked the within-clusters sum-of-squares as well as the average silhouette coefficient for 1 to 10 clusters (Supplementary Figure S4A). Both values decrease with more clusters, but the “elbow” point, or where there is a large reduction in the decrease, was around 4 or 5 clusters. We additionally calculated the individual silhouette coefficients when using 4 clusters to show that each cluster had a significant number of data points with coefficients above the average value (Supplementary Figure S4B).

**Figure 2:**
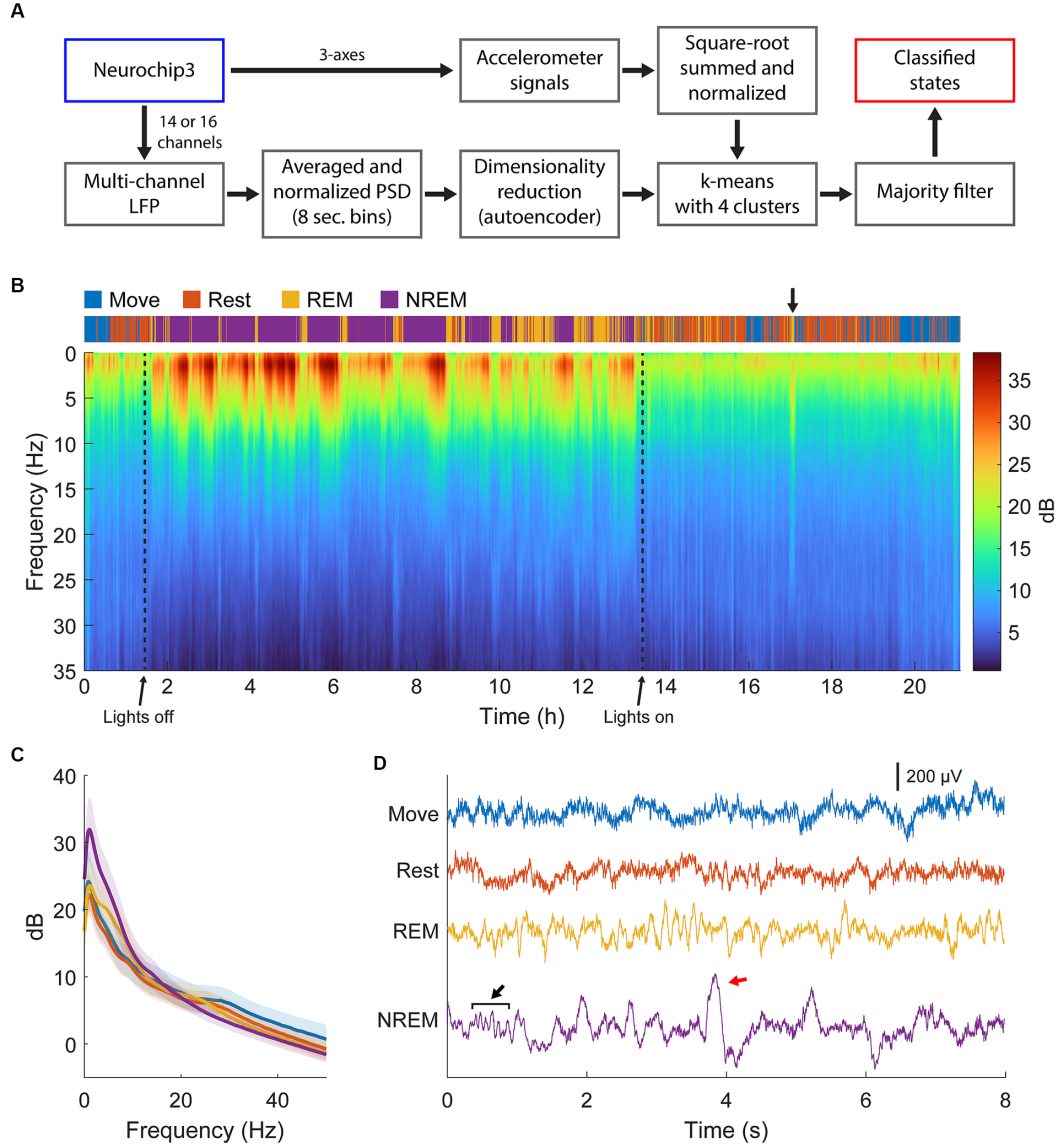
State classification. **(A)** Diagram of the full classification process. Normalized LFP power spectral density was used to train an autoencoder for dimensionality reduction. The lower dimensional representation and normalized accelerometer signals were subsequently clustered with k-means clustering then smoothed with a majority filter to determine different sleep states. **(B)** Example of classification and spectra over 22 h of continuous recording. Brief periods of REM sleep were observed during the day, attributed to naps (arrow). **(C)** Averaged spectra across each state for the session shown in **(B)**. There is high delta power during NREM, theta power during REM, and beta power during Move and Rest states. **(D)** Example traces of raw LFP during each state. Spindles (black arrow) and k-complexes (red arrow) were observed during NREM sleep.

The full classification procedure is outlined in Figure 2A and an example of the final classification over a 21-h period and the corresponding spectral power is shown in Figure 2B. During lights-off we found consistent REM cycles in between NREM epochs occurring every 30 min to 2 h, which became longer and more frequent closer to the morning, consistent with previous findings (Aserinsky and Kleitman, 1953; Kripke et al., 1968; Hsieh et al., 2008). NREM was absent when the lights were on in the animal room, but we often found brief periods of REM sleep concurrent with changes in the PSD, which we attributed to naps (Figure 2B, arrow) (Moses et al., 1975; Dijk et al., 1987). We also often found short periods of Rest or sometimes even Move during the night which were attributed to brief awakenings, typical for a normal night of sleep in non-human primates (Rachalski et al., 2014). An example of averaged spectral power during each sleep state is shown in Figure 2C, and example traces are shown in Figure 2D.

### Sleep state classification was validated to be consistent

**Figure 3:**
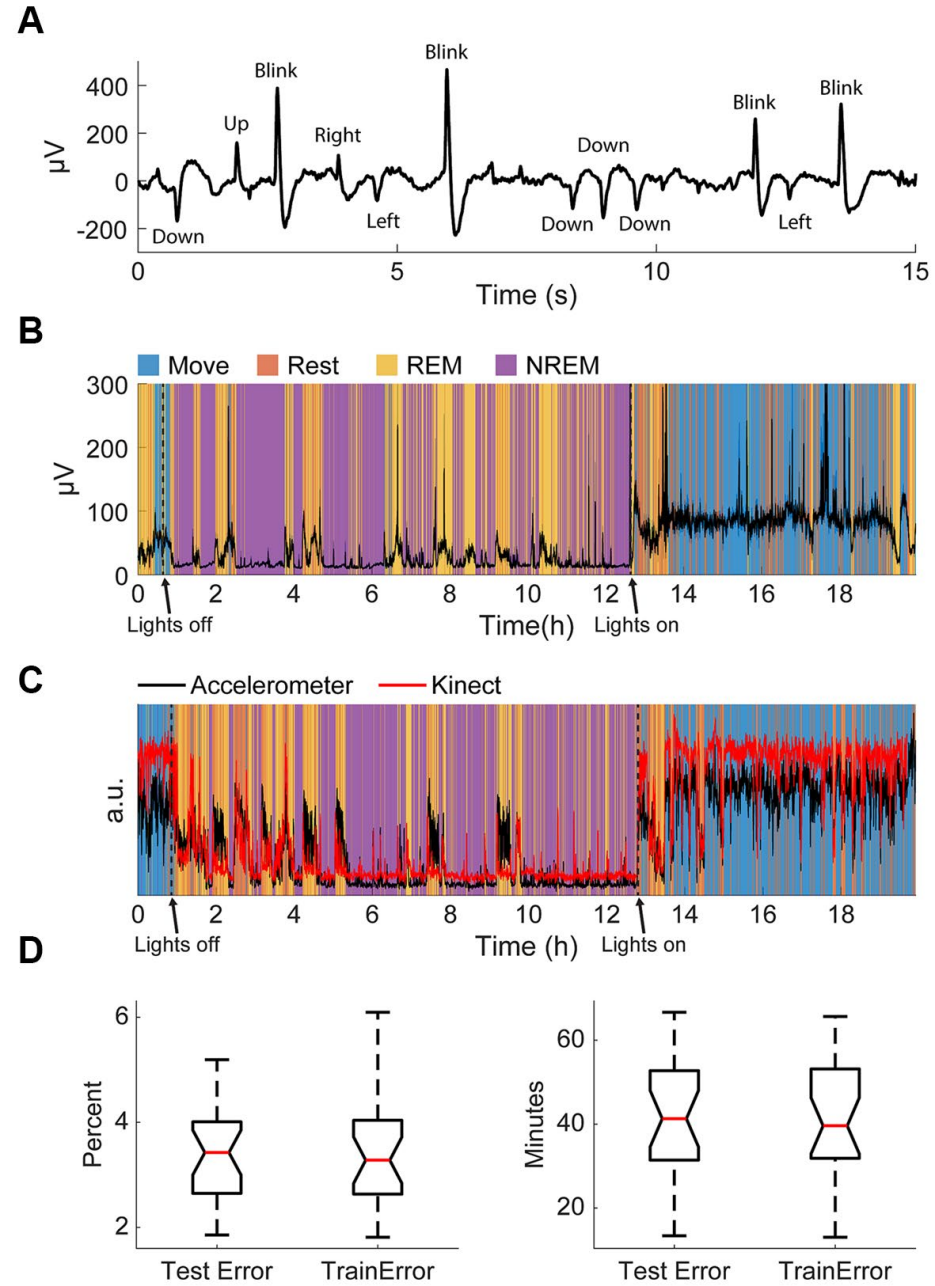
Validation of classification. **(A)** An example of filtered differential EOG signals and corresponding eye movements. **(B)** Standard deviation of EOG signals overnight with respect to classified states. Note the increase in large eye movements during REM and waking. **(C)** An example of the normalized variance of accelerometer data and the normalized variance of the Kinect data with respect to classified states. **(D)** Test and training errors with k-fold cross-validation in percent (left) and total minutes (right).

As our classification scheme was unsupervised, we simultaneously recorded electrooculography (EOG) signals in one animal to validate the identified REM stage during sleep (Figure 3A). Eye movements detected with the EOG were clearly associated with identified sleep states; EOG variance was very high when the animal was awake, very low during NREM sleep, and elevated during REM sleep (Figure 3B). This relationship was present even during very brief windows of detected awakening during the night and naps during the day, indicating high accuracy of state classification.

The accelerometer used for classification was incapsulated within the Neurochip3 which was mounted on the animals’ head. To confirm the accuracy of detected movements, we additionally recorded the monkeys’ movements overnight with a Microsoft Kinect. Compared to the movement values extracted with the Kinect, the accelerometer values were significantly larger during REM sleep (Figure 3C). This is likely due to most movements at night during sleep involving the head. However, these differences were minor, and the two signals were comparable throughout the recording.

Finally, we also performed k-fold cross-validation to verify that our classification method was consistent and robust. In this method, we randomly divided the dataset into k groups then trained the autoencoder for state classification on k-1 groups. The discrepancy between the classification when using k-1 groups from the classification when using all the data for the groups used for training the autoencoder (training error) and the one group that was left out (test error) allows us to determine the consistency of the classification scheme. We used *k* = 20 and carried out 50 repetitions per session and found the average test and training error to be less than 4%, or around 40 total minutes (Figure 3D). The similar but low test and training errors suggest the classification method had low variance (i.e., not overfit) and high consistency. As a result, the classification was deemed appropriate for the aims of this study.

### Changes in LFP power shows changes in oscillatory activity

**Figure 4:**
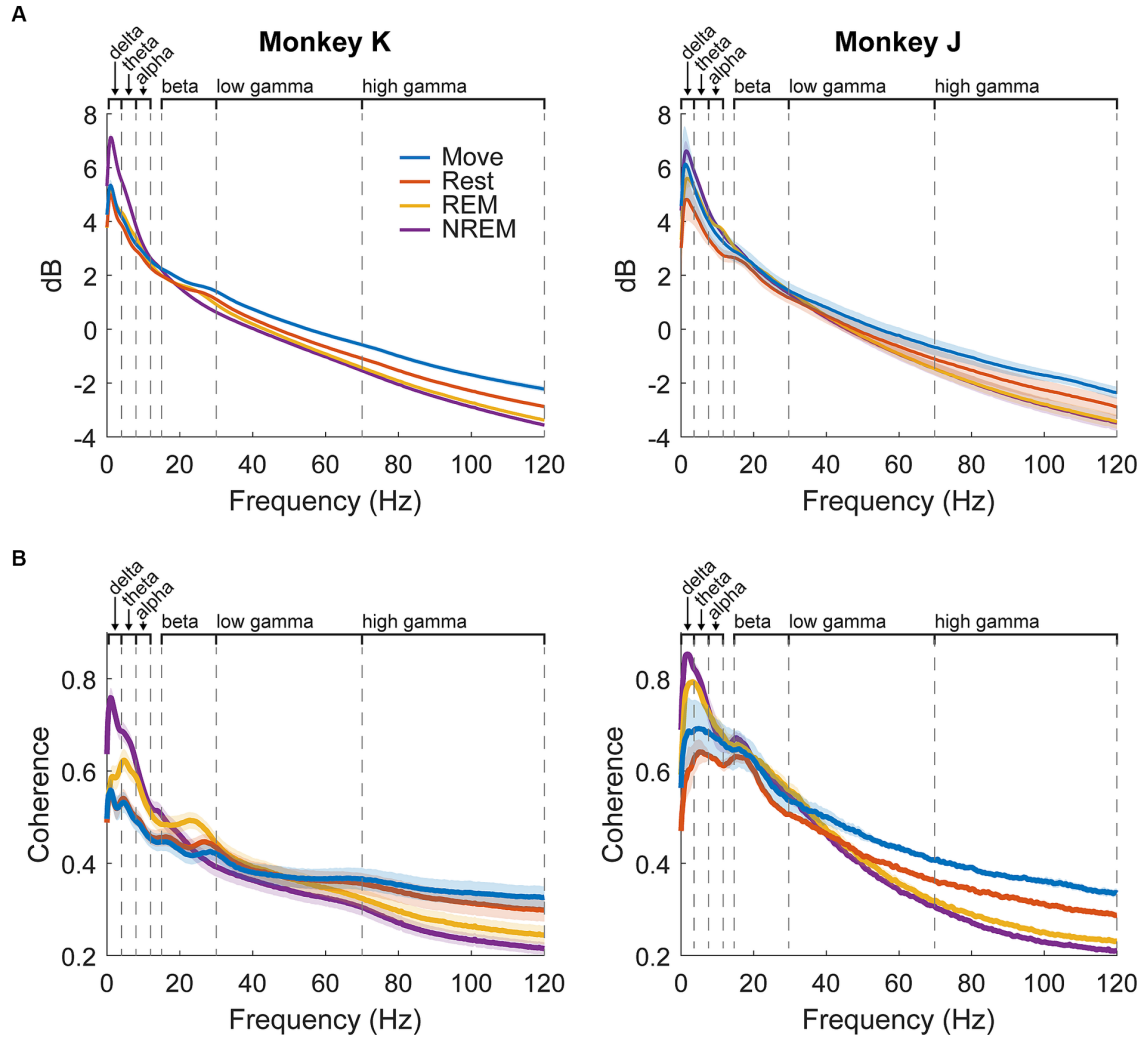
LFP dynamics. **(A)** Average spectral power in each state for each animal across all experiments. Shaded regions show standard error. **(B)** Average pairwise coherence in each state for each animal across all experiments. Shaded regions show standard error.

The average spectral power per state for each animal is shown in Figure 4A. Delta power was high during NREM sleep due to the presence of slow waves. There was a beta peak during the wake states, though the specific frequency range differed between the animals: 25–30 Hz for Monkey K and 15–20 Hz for Monkey J. REM sleep showed slightly higher theta and alpha power compared to the wake states.

Average pairwise coherence per state for each animal is shown in Figure 4B. The features were very similar to those shown in the power, but the differences were magnified, likely due to coherence showing synchrony between pairs of channels and amplifying active oscillatory signaling over baseline spectral density. In addition to the large delta peak during NREM, there was a much clearer difference between REM and the wake states, including a more distinct beta peak. As coherence could be a result of volume conduction, we also measured the weighted phase lag index (wPLI) (Vinck et al., 2011) which showed similar results to coherence (Supplementary Figure S5).

All following cross-frequency phase-amplitude coupling and spike-LFP relationships were combined between the two animals as they demonstrated very similar results as shown in Supplementary Figures S6, S7.

### Cross-frequency phase-amplitude coupling is modulated by brain state

**Figure 5:**
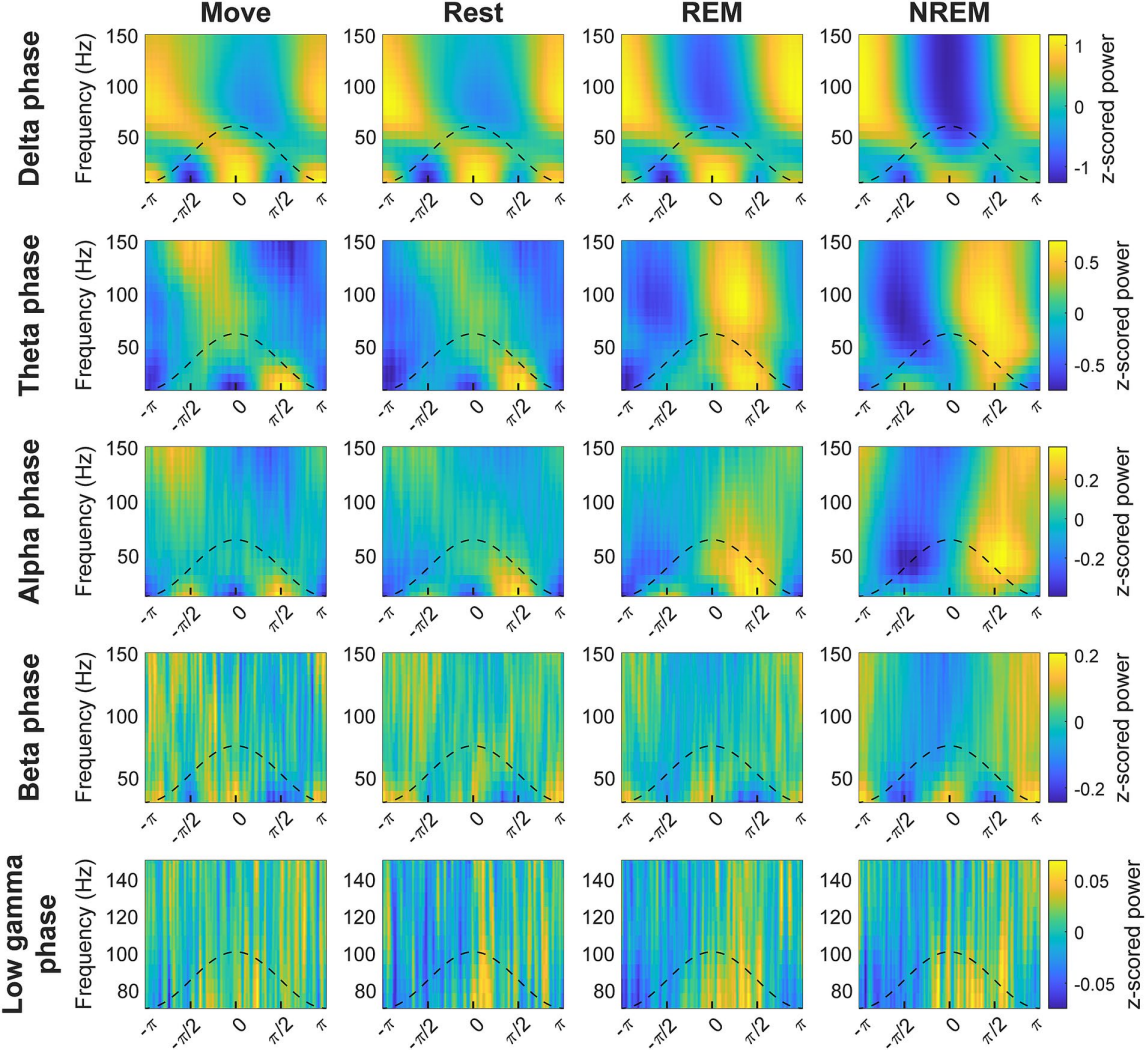
Cross-frequency phase-power distributions. Distribution of lower frequency band phase and higher frequency band power during each state. The power is *z*-scored for each frequency.

The phase of lower frequency bands has been observed to be coupled with the amplitude of higher frequency bands, thought to reflect coordination between brain networks (Jensen and Colgin, 2007; Canolty and Knight, 2010). We explored the coupling between every pair of low frequency band phase to high frequency band amplitude to determine whether there were state-dependent changes. We first plotted the average z-scored spectral power of higher frequencies at different phases of each frequency band (Figure 5). The coupling between different frequency bands in terms of strength and specific phase were modulated by the state of the animal.

**Figure 6:**
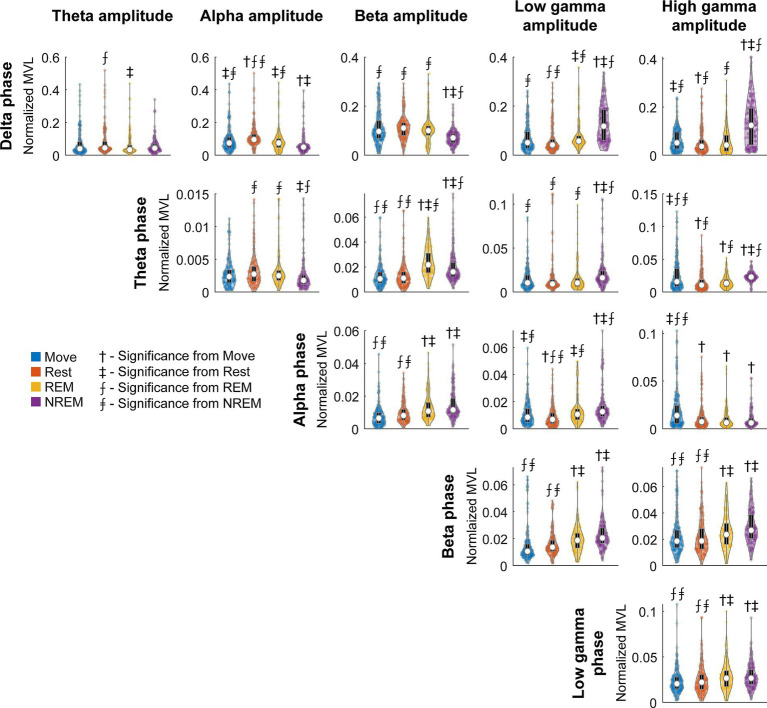
Normalized MVL distributions. Normalized MVL distributions for each lower frequency band phase and higher frequency band amplitude pair during each state. The black boxes show standard box plots with interquartile range and the white dots show median values. The numbers above each group denote significance compared to another state (Friedman’s test, *p* < 0.05).

To quantify the degree of coupling we calculated the normalized mean vector length (nMVL) (Figure 6). We observed state-dependent changes in the distribution of nMVL, most notably (1) high delta phase to alpha amplitude (delta-alpha) coupling during Rest, likely due to increased alpha oscillatory power, (2) low delta-beta coupling during NREM, perhaps for disinhibition of the cortex leading to increased plasticity, (3) high delta-high gamma coupling during Move and NREM, potentially reflecting synchrony of spike activity with delta oscillations, (4) high theta-beta coupling during REM, showing a possible modulation of beta by deeper brain structures, (5) high theta-high gamma coupling during Move, which may reflect hippocampal place cells synchronizing cortical activity during behavior, and (6) high alpha-high gamma coupling during Move, which may be due to the relevance of alpha to both attention and movement preparation. The distribution of mean vector phases for each pair of frequency bands during each state is shown in Supplementary Figure S8.

### Brain states modulate spiking dynamics

**Figure 7:**
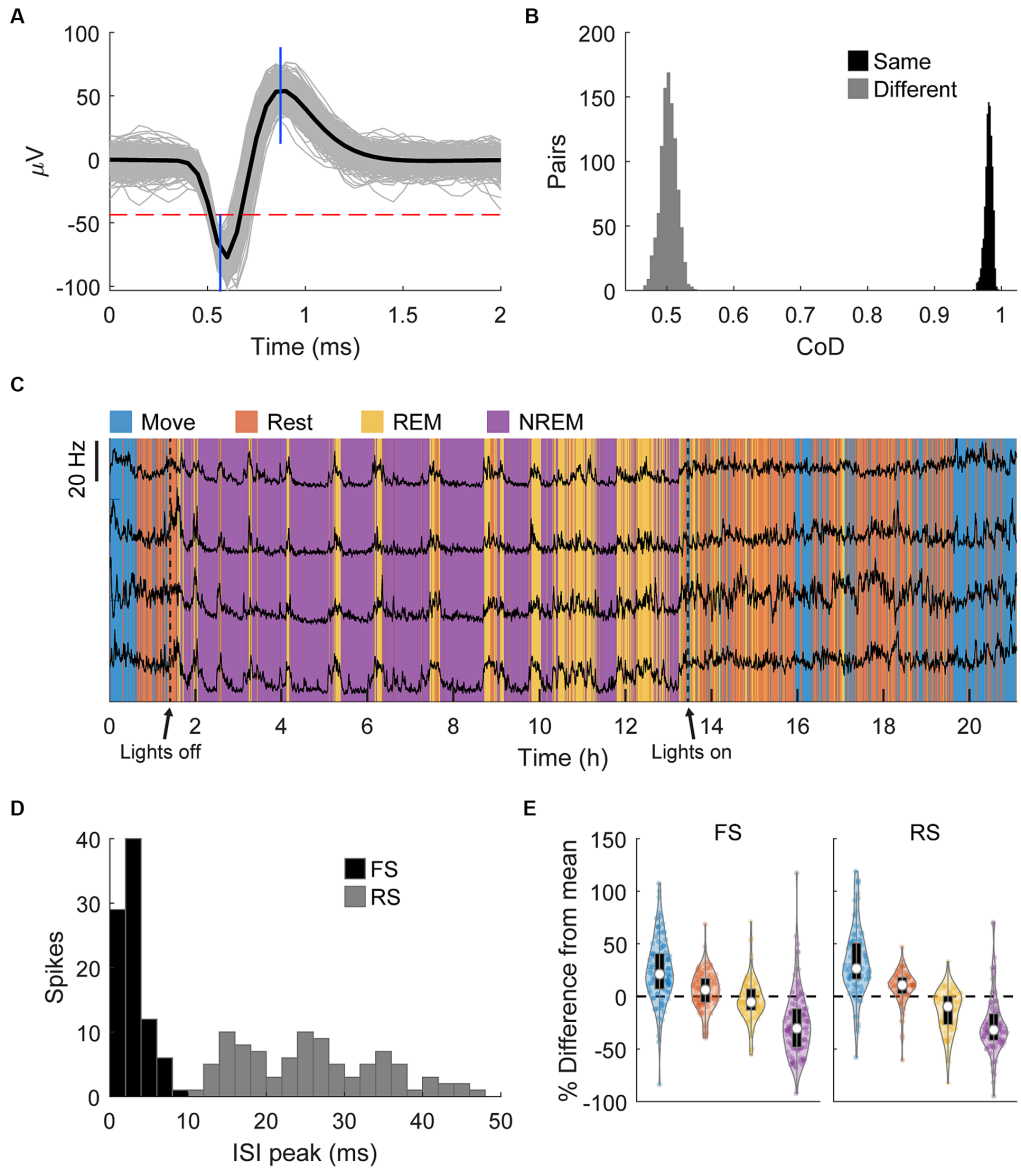
Spike sorting. **(A)** An example of a sorted spike. The gray traces show a random sample of 1,000 spikes, the black line is the average, the horizontal dashed red line shows the threshold, and the blue vertical lines show the two windows. **(B)** An example of comparisons of the pairwise coefficient of determination (CoD) between the first and last 1,000 instances of the same spike and the first and last 1,000 instances of two different spikes. **(C)** Firing rates binned every 60 s of four neurons overnight with classified sleep states. The changes in firing rate very closely match the changes in state. **(D)** Histogram of the peak of the ISI distribution of each spike. Spikes with peaks earlier than 10 ms were denoted to be fast spiking (FS) and all others denoted to be regular spiking (RS). **(E)** Percent difference of firing rate in each state from the average overall firing rate for each spike type. Each state is statistically significantly different from each other state (Friedman’s test, *p* < 0.05).

Spikes were manually sorted using two-window discrimination and confirmed using the coefficient of determination (Figures 7A,B, see *Materials and Methods – Spike sorting*). We tracked a total of 193 spikes (121 in Monkey K, 92 in Monkey J) that satisfied our conditions across all sessions. The firing rates of these single units overnight were strongly and consistently modulated by different identified sleep states, especially during REM cycles during the night, further demonstrating the accuracy of the classification method (Figure 7C). Firing rates were consistently lower with deeper sleep. Assessing the differences in firing rates in individual states showed that both RS and FS firing rates significantly decrease with deeper sleep (Figure 7E).

**Figure 8:**
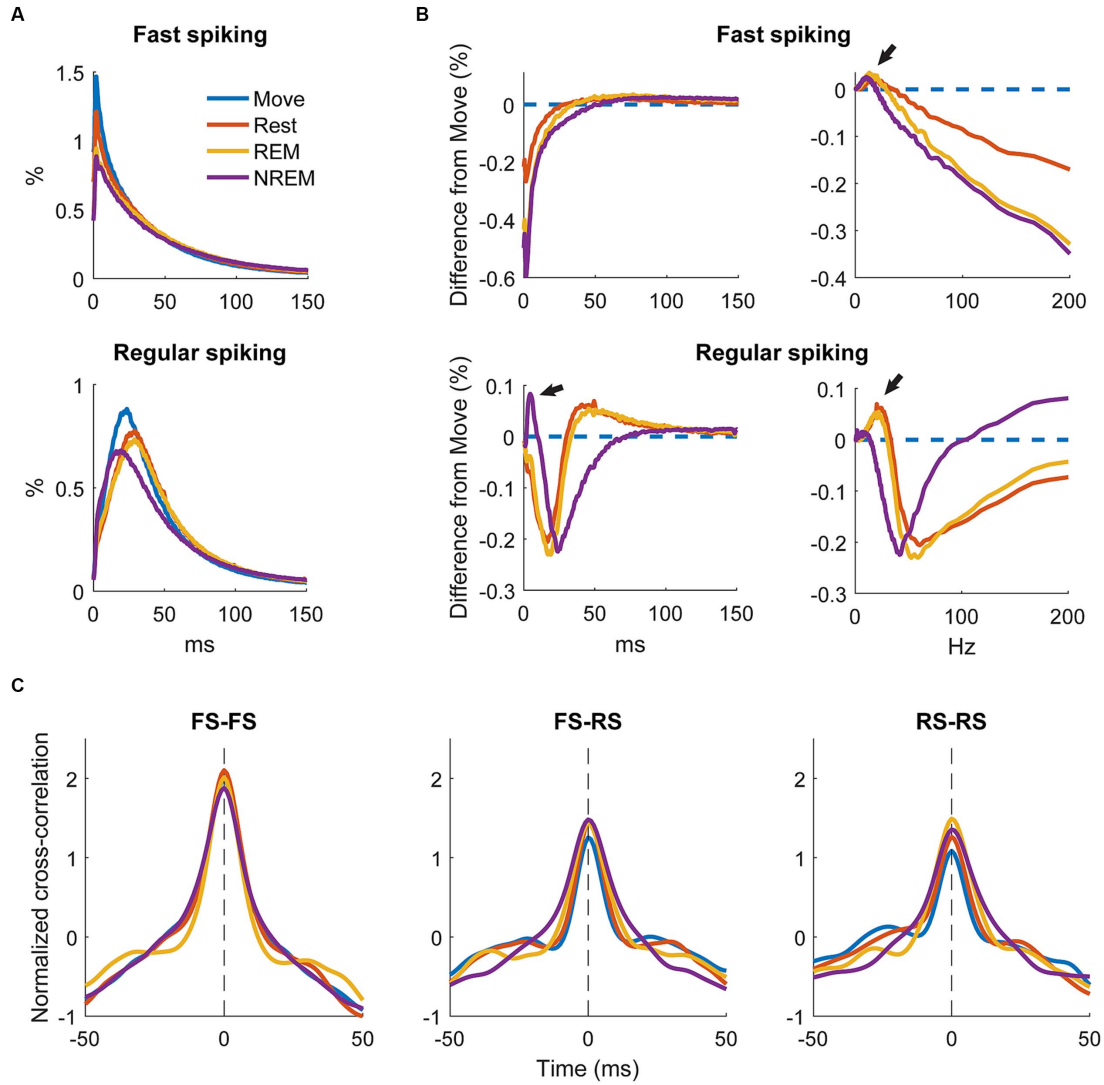
Changes in firing patterns. **(A)** Average ISI distributions of identified fast spiking and regular spiking neurons during each state. **(B)** Difference of ISI distributions from Move plotted against time (left) and frequency (right). Note the peak around 5 ms during NREM for regular spiking neurons and the peaks around 7–10 Hz during NREM sleep and the peaks at around 20 and 25 Hz for REM and Rest, respectively, (arrows) for both neuron types. All three peaks were significantly different from 0 (Wilcoxon signed-rank test, *p* < 0.05). **(C)** Cross correlations of spike firing rates between pairs of fast spiking (FS) neurons (left), FS and regular spiking (RS) neurons (middle), and pairs of RS neurons (right) (*n* = 636, 1,328, 1,036 respectively).

To determine how spiking patterns changed between different states we analyzed changes in the average ISI distributions of RS and FS units. The raw ISI distributions showed an overall decrease on most ISIs with deeper sleep as well as a slight change in the timing of the distribution, especially for RS units (Figure 8A).

To quantify the differences, we calculated the difference in ISI distributions from Move (Figure 8B, left). FS units had a consistent decrease in shorter latency ISIs with deeper sleep, but RS units showed a large increase in short latency ISIs at around 5 ms (arrow). To determine if there were any changes in larger ISIs, we plotted the distributions against frequency (Figure 8B, right). Both FS and RS units showed a peak between 7 and 10 Hz during NREM and a peak between 20 and 25 Hz during Rest and REM.

We additionally sought to determine how the brain state could affect the relationship between units by calculating the cross correlation between firing rates of recorded single units. Firing rates were calculated by convolving the spike train with a gaussian kernel with an approximate width of 10 ms. Figure 8C shows the cross correlations between smoothed firing rates of pairs of FS units, between FS and RS units, and between pairs of RS units. There was not a clear difference in the cross correlation between the four states suggesting relative circuitry is maintained. The correlation between pairs of FS units was stronger than between FS and RS or pairs of RS units, as inhibitory neurons are often more interconnected within the cortex.

### Brain states modulate spike-field dynamics

**Figure 9:**
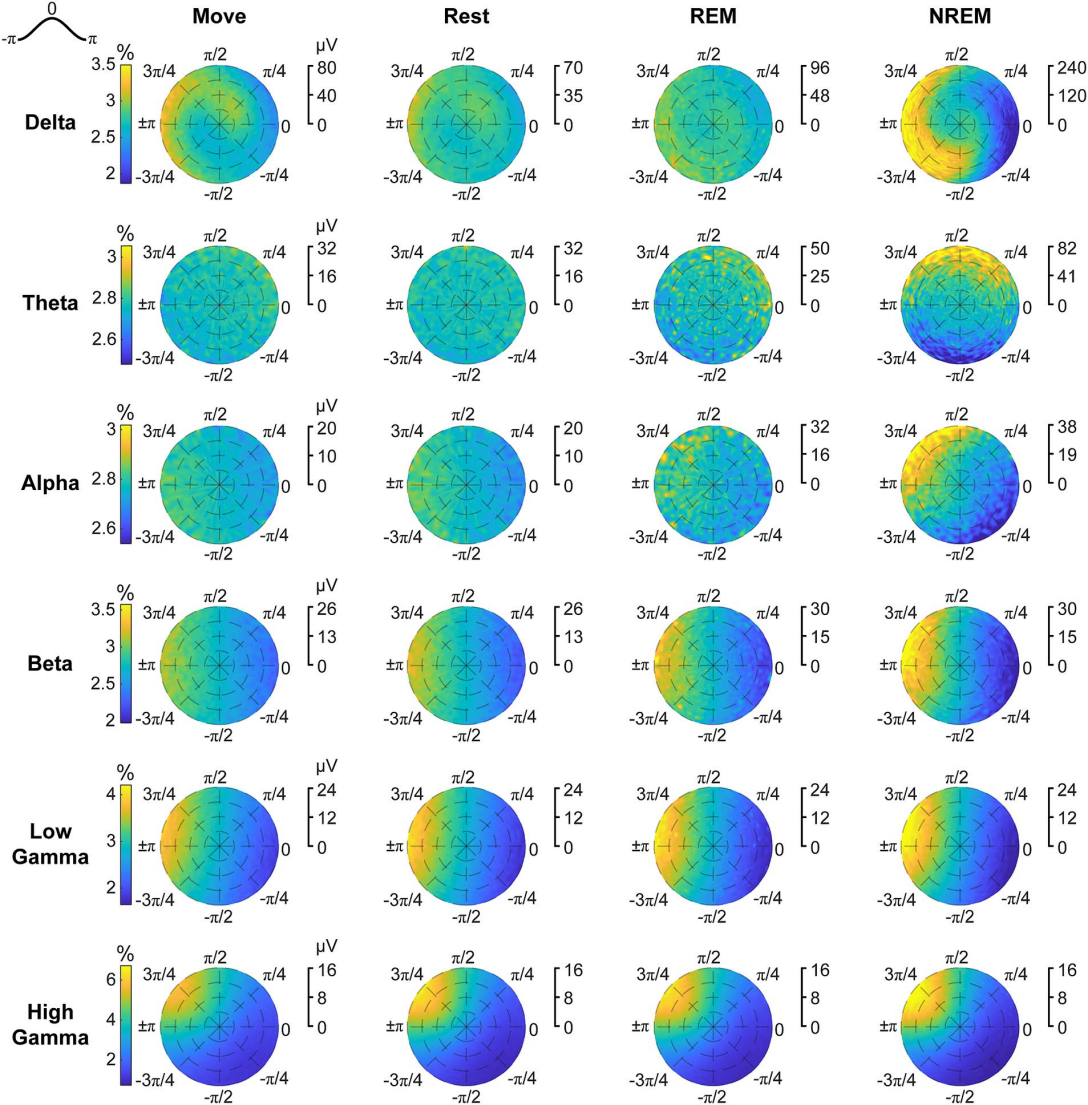
Phase distributions of LFPs at spike times. Phase distributions normalized for each amplitude during each sleep state for each LFP band.

Spikes have been shown to be synchronized to various frequency bands, particularly in the motor cortex (Murthy and Fetz, 1996b; Buzsáki et al., 2012). We first analyzed the phase-amplitude distribution normalized by amplitude of each LFP band during spike timings (Figure 9). There was clear synchrony with spikes during NREM sleep with all LFP bands, though at different phases. During NREM spikes typically occurred at the trough of delta, beta, and gamma activity, and at intermediate phases for theta ( ~π/2 ) and alpha ( ~3π/4 ). The synchrony was particularly apparent during high amplitudes, suggesting spikes are synchronized to active frequency band activity rather than epiphenomena arising from periodicity of spike firing patterns. During both awake states and REM sleep, spikes were synchronized to the trough of beta and gamma frequencies, though less than during NREM sleep. Spikes during Move were also potentially synchronized to the delta band. All other combinations did not show apparent coordination between LFP band phase and spike timing.

**Figure 10:**
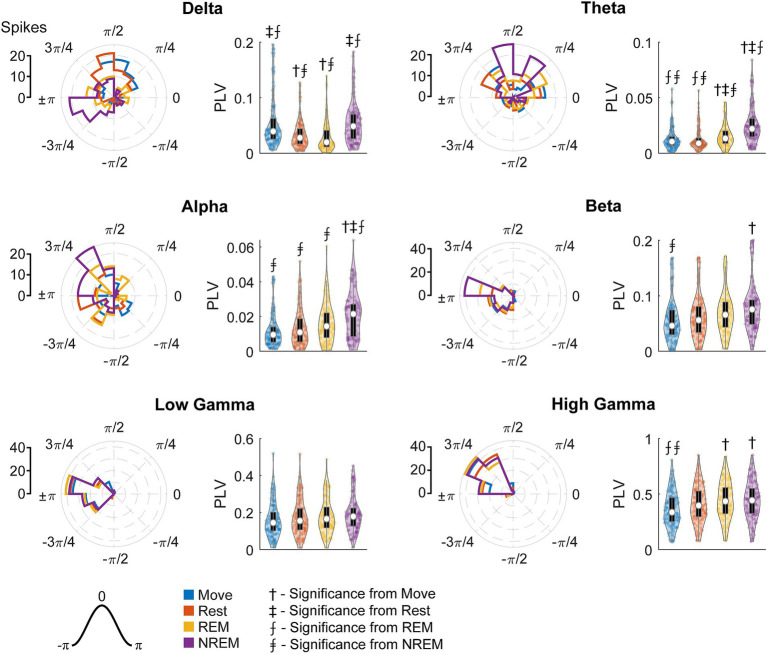
Locked phase and phase locking value distributions for regular spiking neurons. Distributions of locked phase (left) and PLVs (right) of regular spiking neurons during each sleep state for each LFP band. The numbers above each group denote significance compared to another state (Friedman’s test, *p* < 0.05).

To quantify the strength of these relationships, we calculated the phase-locking value (PLV) for each unit during each state for all LFP bands. Figure 10 shows the RS unit spikes’ distribution of locked phases as well as the PLV for each frequency band during each state. Spikes were more synchronized to delta during both Move and NREM, although the preferred phases are different: π/2 for Move and ±π for NREM sleep. For every other frequency band, there was stronger synchrony during deeper sleep states, though the differences were often not statistically significant. The preferred phases were extremely consistent across states for beta and higher frequency bands peaking between ±π and 3/4π, but variable for alpha and theta bands which also showed lower PLVs across all states.

**Figure 11:**
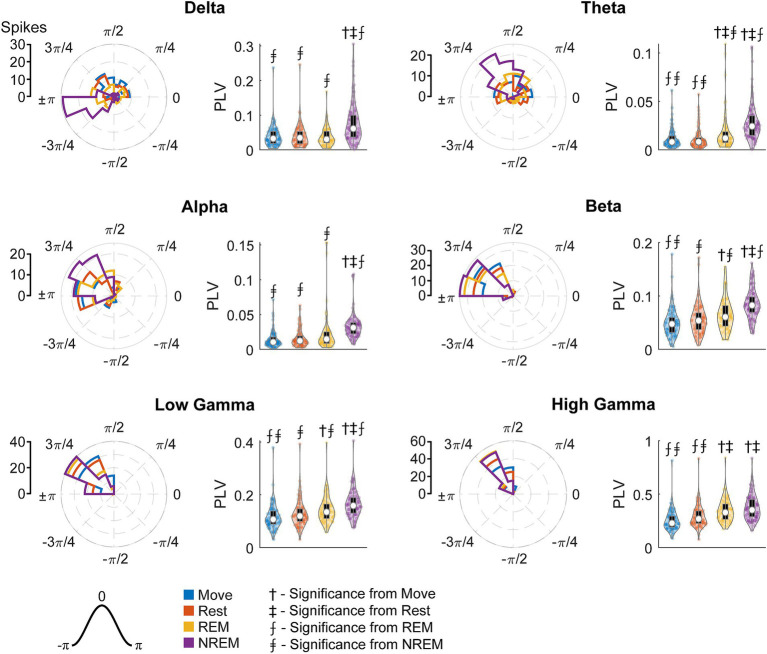
Locked phase and phase locking value distributions for fast spiking neurons. Distributions of locked phase (left) and PLVs (right) of fast spiking neurons during each sleep state for each LFP band. The numbers above each group denote significance compared to another state (Friedman’s test, *p* < 0.05).

Figure 11 shows the FS unit spikes’ distribution of locked phases and the PLV for each frequency band during each state. One large difference from RS units was that FS units were synchronized to delta oscillations during Move. As opposed to RS units, FS units also showed significantly larger PLVs during NREM than other states for all frequency bands. In addition, the phase of synchrony to the low and high gamma bands was earlier in the cycle for FS units compared to RS units. However, the phase of synchrony to delta oscillations during NREM for both RS and FS units were both around the trough (π).

## Discussion

### Brain states were consistently classified and validated

Classification of sleep states is often an inconsistent and arduous task. Criticisms of and modifications to the guideline (Himanen and Hasan, 2000; Silber et al., 2007) as well as various automated methods (Penzel and Conradt, 2000; Hamida and Ahmed, 2013; Sun et al., 2020) have been proposed, but each approach differs in the set of defined states and the type and amount of data required. In addition, studies on sleep typically use electroencephalogram (EEG) recordings, which are distinctly different from intracortical LFPs due to their lower spatial specificity and the complex frequency and phase filtering of bone and tissue, especially at higher frequencies (Michel et al., 2004; Buzsáki et al., 2012; Sejnowski et al., 2014). Beyond these difficulties, manual scoring is also compromised by high inter-scorer variability (Himanen and Hasan, 2000; Younes et al., 2016).

As a result, we developed our own classification methodology tailored to our data to distinguish between different sleep states. The process utilizes a stacked-autoencoder for dimensionality reduction followed by clustering on processed LFP PSDs and accelerometer values. We used several metrics to validate the method: EOG recordings, concurrent recordings with the Kinect to track movement, k-fold cross validation, and changes in spike rates; they all indicated accurate and robust classification (Figure 3). This accuracy may be the result of the high spatial specificity of our recording probe focused on a small area (4 × 4 mm) of cortex; previous studies have shown that sleep can be local and vary between different regions of the brain (Vyazovskiy et al., 2011; Mascetti, 2016; Siclari and Tononi, 2017).

Our method could potentially be improved by using a dimensionality reduction method with higher complexity such as a deep neural network (Malafeev et al., 2018), different clustering methods such as hierarchical clustering (Gerla et al., 2019), and by including the EOG recordings or other biophysical measurements as additional dimensions of data such as EMG (Estrada et al., 2006; Khalighi et al., 2013). However, developing a flawless classification method was not within scope of the study and the method presented was deemed to be sufficient.

### Spike dynamics vary due to different sleep states

We observed decreased spiking activity with deeper sleep, consistent with previous studies (Figure 7; Steriade et al., 2001; Xu et al., 2019). In addition, the firing patterns of units changed due to sleep state – RS units had a relative increase in low ISI activity during NREM compared to Move, and both RS and FS units had relative increase in high ISI activity during Rest, REM, and NREM compared to Move.

We also observed an increase in beta frequency activity of units during REM and Rest (Figure 8) which is consistent with the proposal that beta reflects a “resting” rhythm in the primary motor cortex (Engel and Fries, 2010). RS units are likely to be excitatory pyramidal cells and FS units are likely to be inhibitory interneurons (McCormick et al., 1985), which may explain the relative increase of short ISIs in RS units during NREM sleep: NREM has often been tied with reactivation of cortical circuitry which may be driven by excitatory neurons.

### Cross-frequency coupling and spike-LFP synchrony suggest delta and theta may reflect mechanisms of sleep

Cross-frequency coupling refers to modulation of a higher frequency band by a lower frequency band (Figure 1C). Such coupling reflects coordination of local networks operating on shorter time scales (i.e., low and high gamma activity) to distributed circuits synchronized at longer time scales (i.e., delta activity). These relationships could potentially play a role in neural mechanisms of attention, learning, and memory (Jensen and Colgin, 2007; Canolty and Knight, 2010). Compared to other proposed measures to quantify phase-amplitude coupling, the MVL introduced by Canolty et al. (2006) has been shown to be accurate, the most sensitive to modulations in coupling strengths, and ideal for high quality signals over long recording epochs (Canolty et al., 2006; Tort et al., 2010; Onslow et al., 2011; Hülsemann et al., 2019).

Spike-LFP synchrony provides another measure of synchrony between the single units and the composite synaptic activity of the local population (Murthy and Fetz, 1996b; Okun et al., 2010; Buzsáki et al., 2012). The magnitude indicates the strength of synchrony whereas the phase reflects the timing of the spikes relative to the local population. We focused on 6 different frequency bands commonly delineated in the cortex: (1) delta (0.5–4 Hz), (2) theta (4–8 Hz), (3) alpha (8–12 Hz), (4) beta (15–30 Hz), (5) low gamma (30–70 Hz), and (6) high gamma (70–120 Hz).

Most instances of cross-frequency phase-amplitude coupling and spike-LFP synchrony were stronger for deeper sleep states. This may be due to asynchronous activity during Move being driven by functional local circuitry (i.e., generating movement) whereas activity during resting and sleep states are more attuned to baseline macroscopic rhythmic activity, potentially related to homeostatic plasticity (Tononi and Cirelli, 2014).

Of the many changes we observed, two were of particular interest – delta-high gamma coupling during Move and theta-beta coupling during REM – due to their unexpected properties and possible implications.

#### Delta and high gamma

Delta band LFP has been shown to be linked to cognitive processing throughout the brain (Güntekin and Başar, 2016). Some evidence suggests delta in the motor cortex is relevant to movement preparation (Saleh et al., 2010; Körmendi et al., 2021) or top-down attention processes (Morillon et al., 2019) through thalamocortical circuitry, but delta in the motor cortex has not been commonly studied. In contrast, high gamma band LFP is well accepted to be representative of local spiking activity, often strongly correlated with action potentials (Ray et al., 2008; Ray and Maunsell, 2011).

As such, the existence of delta-high gamma coupling while the animal was awake was unexpected. The coupling was strong even when separately calculated for each animal (Supplementary Figure S9). Andino-Pavlovsky et al. (2017) showed high delta-high gamma coupling during slow wave sleep in the rodent prefrontal cortex, and Takeuchi et al. (2015) showed strong delta-high gamma coupling during REM and several NREM substages in the primate hippocampus (Takeuchi et al., 2015; Andino-Pavlovsky et al., 2017). However, neither study pursued the state-dependent comparisons, and their broad definition of gamma (>25 Hz) makes independent interpretation difficult. Specific comparisons of delta-high gamma coupling during different sleep states have not been reported.

Spike synchrony to delta band potentially sheds light on the delta-high gamma relationship. In our study, only RS units showed higher delta-high gamma coupling during Move, and the phase of synchrony was consistently located at the falling edge of the wave (Figure 10), or right before the greatest depolarization. During NREM both RS and FS units were synchronized to delta, but the phase of synchrony was immediately after the trough of the wave, or right after the greatest depolarization. Since delta is indicative of macroscopic activity, this suggests that spikes during the day may drive the activity whereas the spikes are driven by the activity at night. Such a relationship potentially reflects delta as indicative of coordinating the cortical circuitry that was engaged during the day for reactivation during sleep, a commonly studied mechanism present in NREM sleep (Ramanathan et al., 2015; Gulati et al., 2017; Xu et al., 2019).

#### Theta and beta in the motor cortex during REM sleep

Theta oscillations have often been observed during REM sleep and have also been shown to coordinate hippocampal place cell activity during active exploration (Cantero et al., 2003; Buzsáki and Moser, 2013). Theta has also been shown to be coupled to low gamma. A wealth of recent cross-frequency coupling literature has reported theta-low gamma coupling in both hippocampus and the neocortex, showing modulations related to task performance, cognitive engagement, and memory formation (Canolty et al., 2006; Buzsáki and Wang, 2012; Lisman and Jensen, 2013). Although stronger theta-low gamma coupling has also been observed during REM sleep, human studies have shown delta-low gamma coupling potentially because hippocampal theta oscillations are slower in humans than in rodents (Cantero et al., 2003; Clemens et al., 2009; Jacobs, 2014). However, we did not find significant delta- or theta-low gamma coupling during REM in non-human primates.

Instead, we found very high theta-beta coupling during REM sleep. Beta is theorized to be indicative of a regulating, inhibitory rhythm in the motor cortex (Engel and Fries, 2010; Kilavik et al., 2013; Khanna and Carmena, 2017), and our results show a general increase in beta power during REM sleep (Figure 4B). However, theta-beta coupling is not commonly studied; some evidence suggests that it plays a role in working memory and decision making, but the literature is sparse and focuses on the frontal lobe (Cohen et al., 2009; Axmacher et al., 2010; Liang et al., 2021). In our analysis, the maximum beta amplitude occurs right before the peak of theta (Supplementary Figure S8), which means minimum beta occurs right after the trough of theta This suggests theta may reflect disinhibition that increases the excitability of the cortex by minimizing beta. Such changes in excitability may then lead to more effective memory consolidation often seen during REM sleep (Boyce et al., 2016); however, more research is needed to confirm these speculations.

### Limitations of the study

Although the limited region of the cortex we recorded aided in our ability to classify different brain states, it also limited our ability to extend the interpretation of our results to the broader functions involved with sleep. Simultaneous recordings of deep brain structures or even different cortical regions may provide more insight into the roles behind the relationships between LFP bands and spikes that were uncovered in this study.

## Conclusion

Our study provides the first comprehensive analysis of cross-frequency phase-amplitude coupling and spike-field synchrony across all frequency bands within the macaque motor cortex during different behavioral states. We observed an increase in short ISIs and high coordination between spikes during NREM, consistent with previous findings suggesting reactivations of cortical circuity during NREM. High cross-frequency phase-amplitude coupling between delta and high gamma when the animal is awake and moving and during NREM sleep, as well as spike-field synchrony with delta band LFP during those states, suggest that delta may be associated with encoding and subsequently driving these reactivations. Previously seen modulations in delta or theta to low gamma phase-amplitude coupling, was not observed. Instead, we observed high theta to beta coupling during REM, potentially reflecting the role of theta during REM sleep as a disinhibiting signal. These results support previous findings and may serve as the basis for future studies into the roles of LFP frequency bands and different sleep states.

## Data availability statement

The raw data supporting the conclusions of this article will be made available by the authors, without undue reservation.

## Ethics statement

The animal study was approved by University of Washington Institutional Animal Care and Use Committee. The study was conducted in accordance with the local legislation and institutional requirements.

## Author contributions

RY: Conceptualization, Data curation, Formal analysis, Funding acquisition, Investigation, Methodology, Project administration, Resources, Software, Validation, Visualization, Writing – original draft, Writing – review & editing. IR: Conceptualization, Investigation, Methodology, Resources, Supervision, Validation, Writing – review & editing. SP: Conceptualization, Funding acquisition, Project administration, Resources, Supervision, Validation, Writing – review & editing. RR: Supervision, Validation, Writing – review & editing. EF: Conceptualization, Funding acquisition, Project administration, Resources, Supervision, Validation, Writing – review & editing.
