# Supplementary material for: Local field potentials and single unit dynamics in motor cortex of unconstrained macaques during different behavioral states
Source: Front Neurosci. 2023 Nov 23;17:1273627. doi: 10.3389/fnins.2023.1273627 (PMC10702227; doi:10.3389/fnins.2023.1273627)

Supplementary Figure S1. Spike features. A. Spike width, dV/dt ratio (the fastest rise of the spike divided by the fastest fall of the spike as defined in McCormick et al. 1985), and the average firing rate with respect to the peak of the inter-spike interval (ISI) distribution. The spike widths include the addition of a random jitter sampled from a uniform distribution from -0.02 to 0.02 ms to better visualize the data. The dashed vertical line denotes the boundary between fast- and regular-spiking neurons (FS and RS respectively) as defined in this study. **B.** Box plots of the corresponding measures and the statistical significance between the two spike types (Wilcoxon rank-sum test). The two measures reflecting the spike shape have significant differences. Although FS neurons have a wider spread of average firing rates, there is no significant difference between the average firing rates of the two types of neurons.

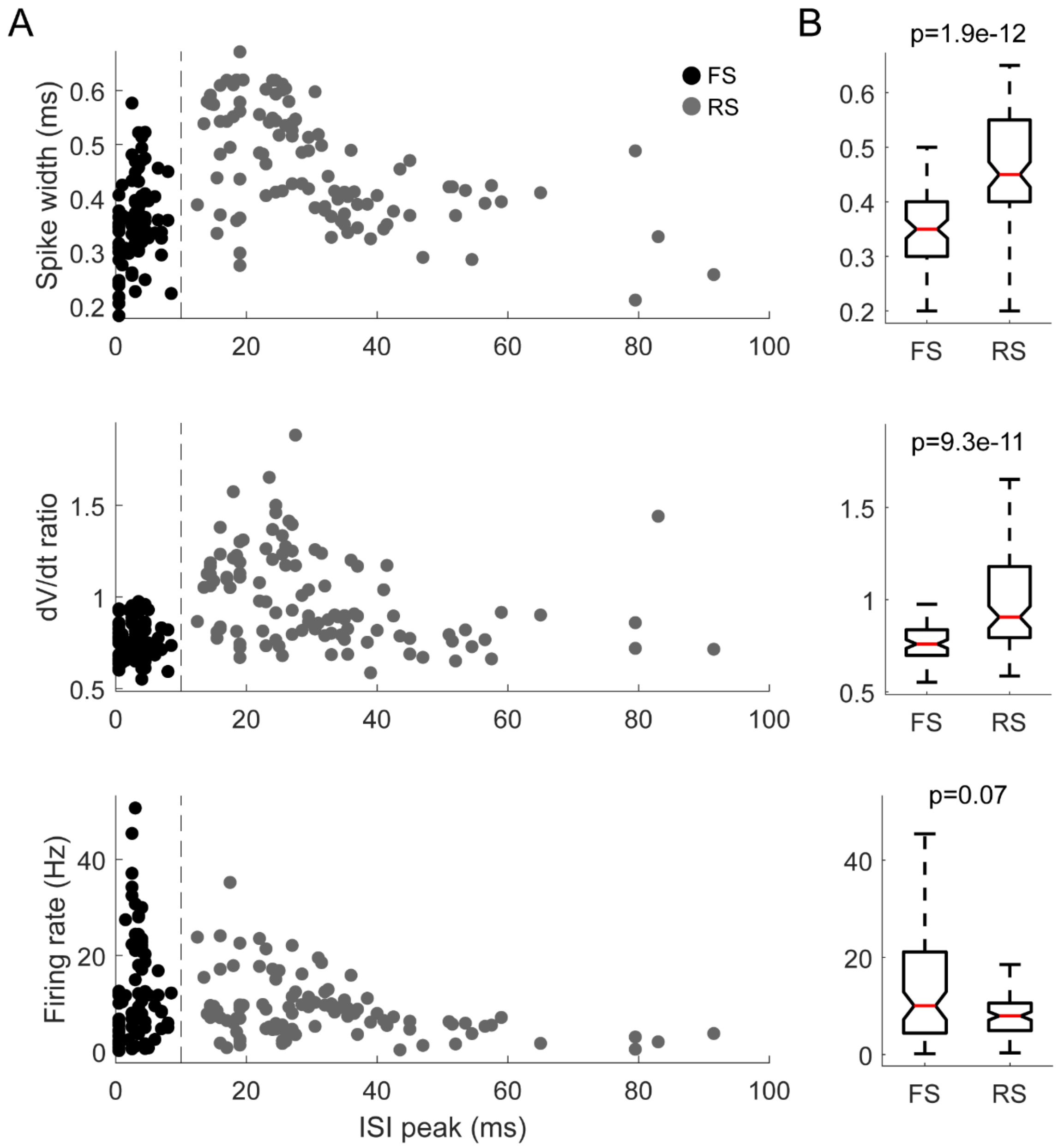

Supplementary Figure S2. Effect of number of spikes on phase locking value. A. A histogram showing the number of spikes per state for each single unit per session. 98% of all instances have greater than 10,000 spikes. **B.** To determine the effect of the number of spikes used to calculate the PLV, we bootstrapped (n=1000) varying numbers of phases from a) a randomly generated phase from a uniform distribution and b) real spike synchronized data. The p-value of the difference between the two samples are shown as the dashed line (Wilcoxon rank-sum). The two samples are significantly different even at 100 spikes (p=0.047), and the real data shows a clear plateau of PLV beginning around 10,000 spikes. Note the p-value is calculated to be 0 beyond 10,000 spikes due to floating point precision. **C.** Difference between different number of samples of real data shows that the calculated PLV value does not change significantly after 5,000 spikes (Kruskal-Wallis, p<0.05). The consistency shows that the effect of sampling bias becomes negligible with higher numbers of spikes.

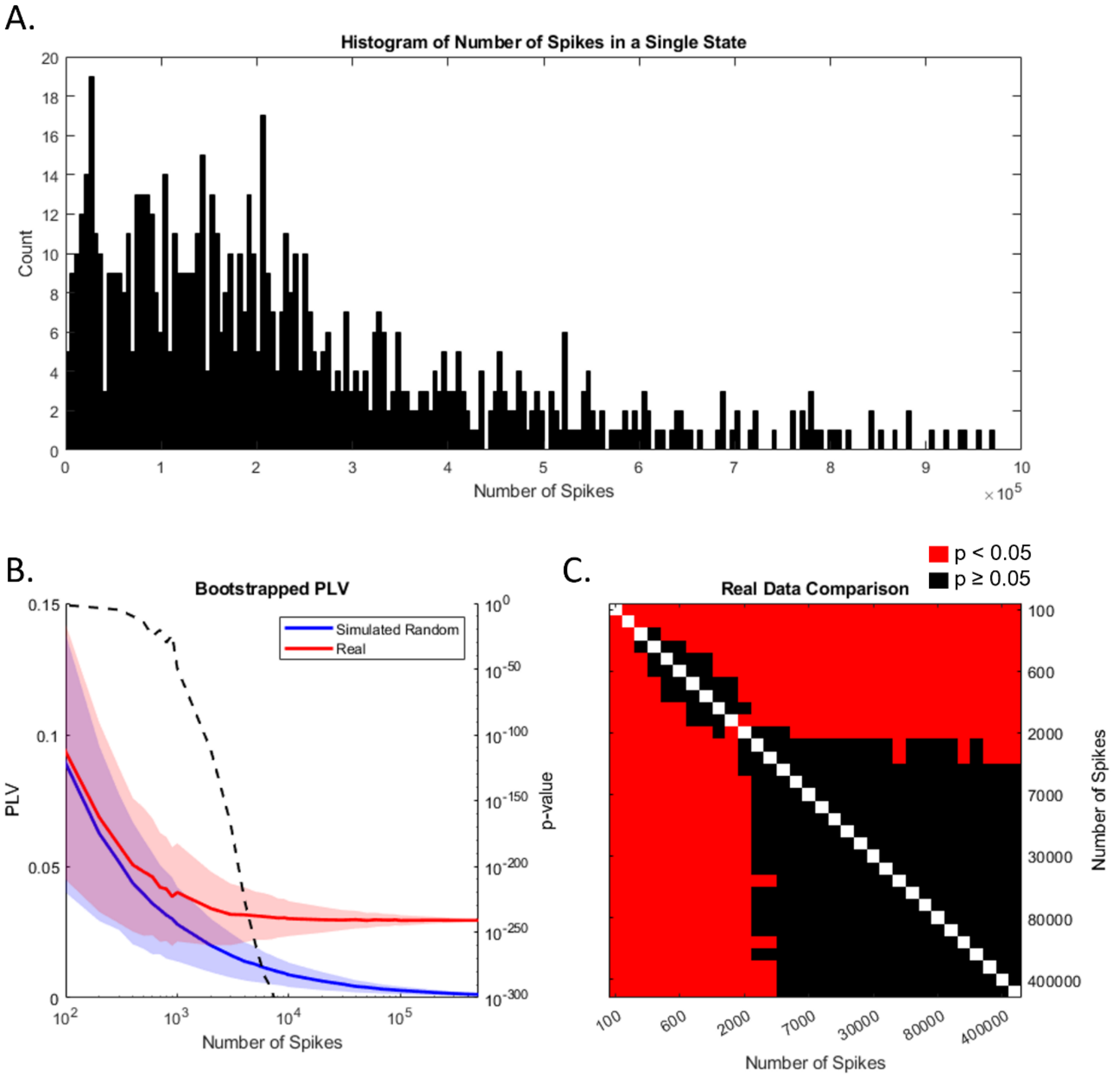

Supplementary Figure S3. Dimensionality Reduction. A. Examples of original spectra and the reconstruction at the output of a trained autoencoder for each classified state. **B.** t-SNE visualization of the low dimensional representation with finalized states. **C.** An example of the average of 100 spectra that generated the highest values in each hidden unit. The darkness of the traces is weighted by the variance in the values of all values in that hidden unit (i.e. more relevant during clustering). **D.** The average of 100 spectra that generated the lowest values in each hidden unit subtracted from (C).

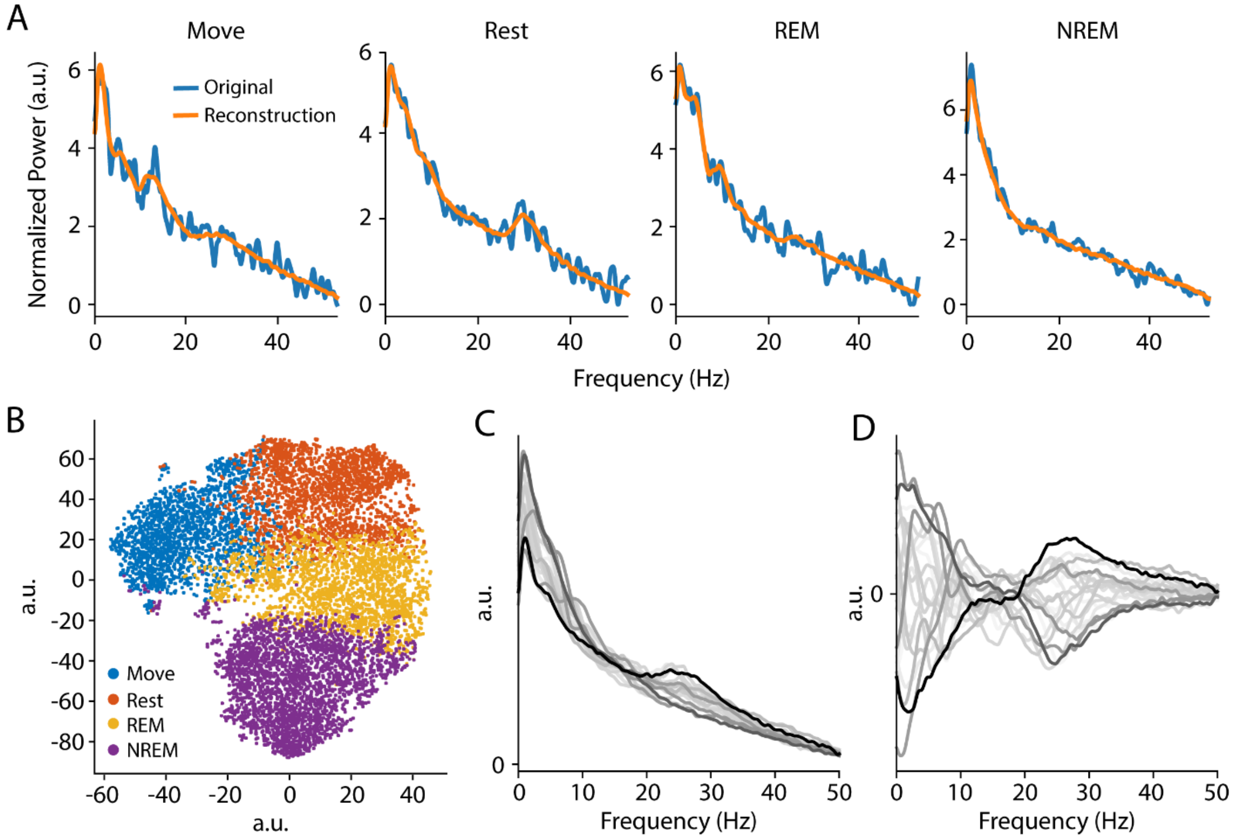

Supplementary Figure S4. K-means clustering. A. The average inertia (black), or within-clusters sum-of-squares, and the average silhouette coefficient (red) of the low dimensional data for different number of clusters. **B.** The silhouette coefficient of each data point with the final classifications compared to the average (vertical red line). All clusters have significant portions above the average.

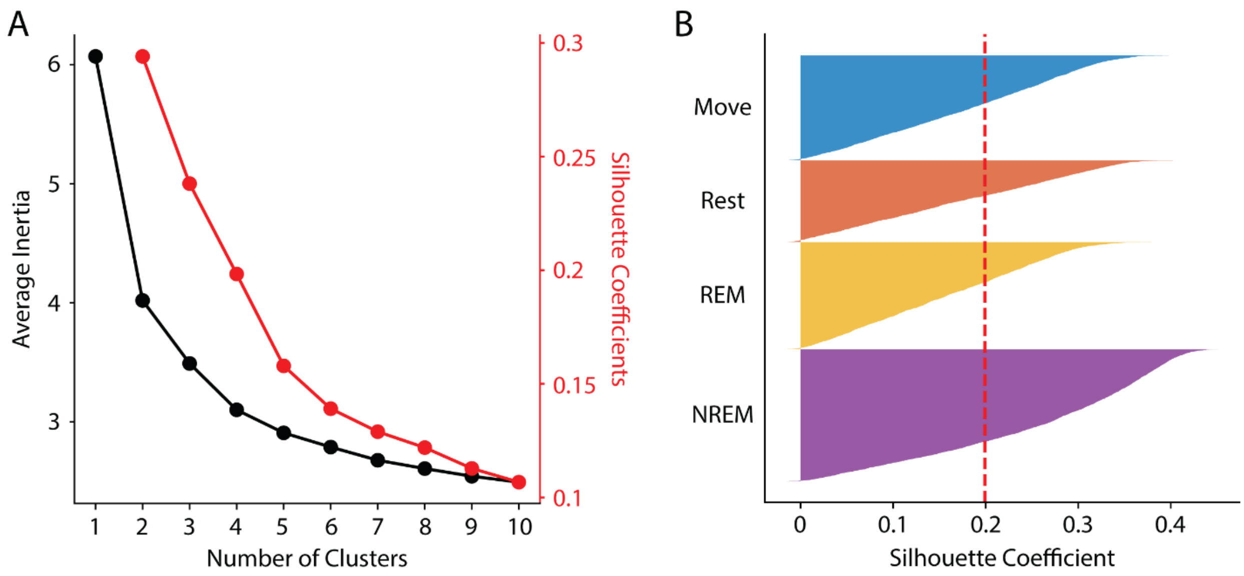

Supplementary Figure S5. Average weighted phase lag index (wPLI). Separated for each animal between the four states. Shaded regions show standard error.

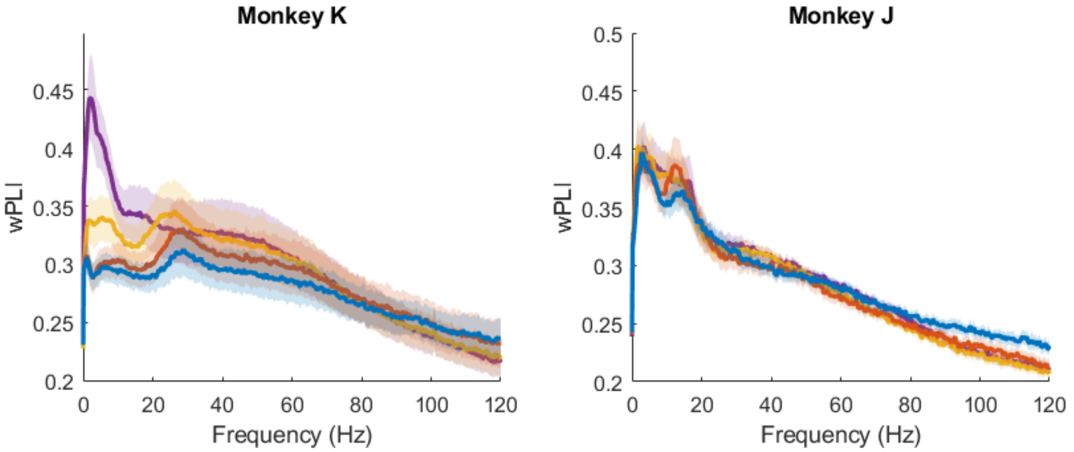

Supplementary Figure S6. Normalized MVL distributions. Normalized MVL distributions for each lower frequency band phase and higher frequency band amplitude pair during each state separated for each animal. This figure corresponds to Figure 6 in the main body of the manuscript.

Monkey K

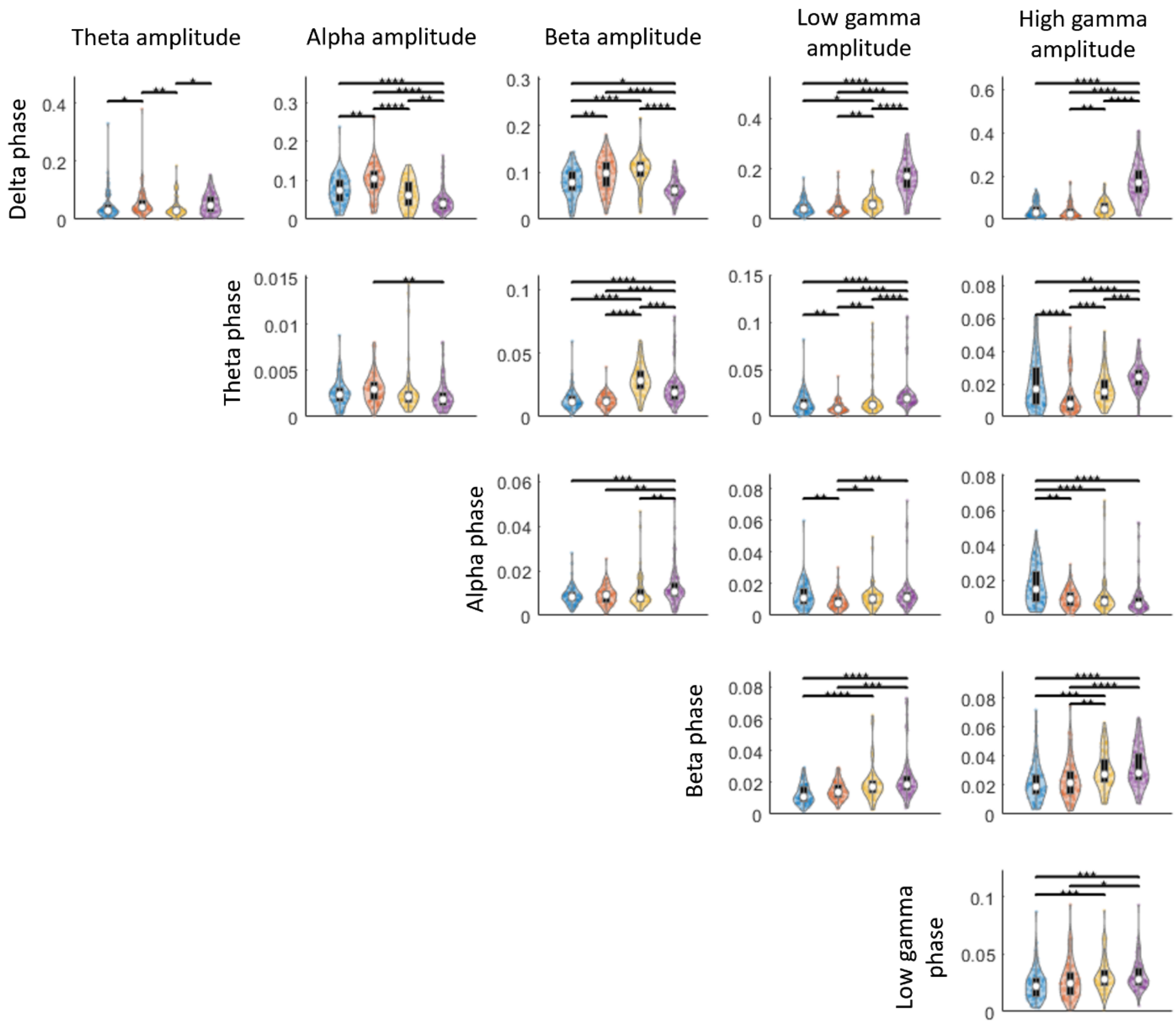

Monkey J

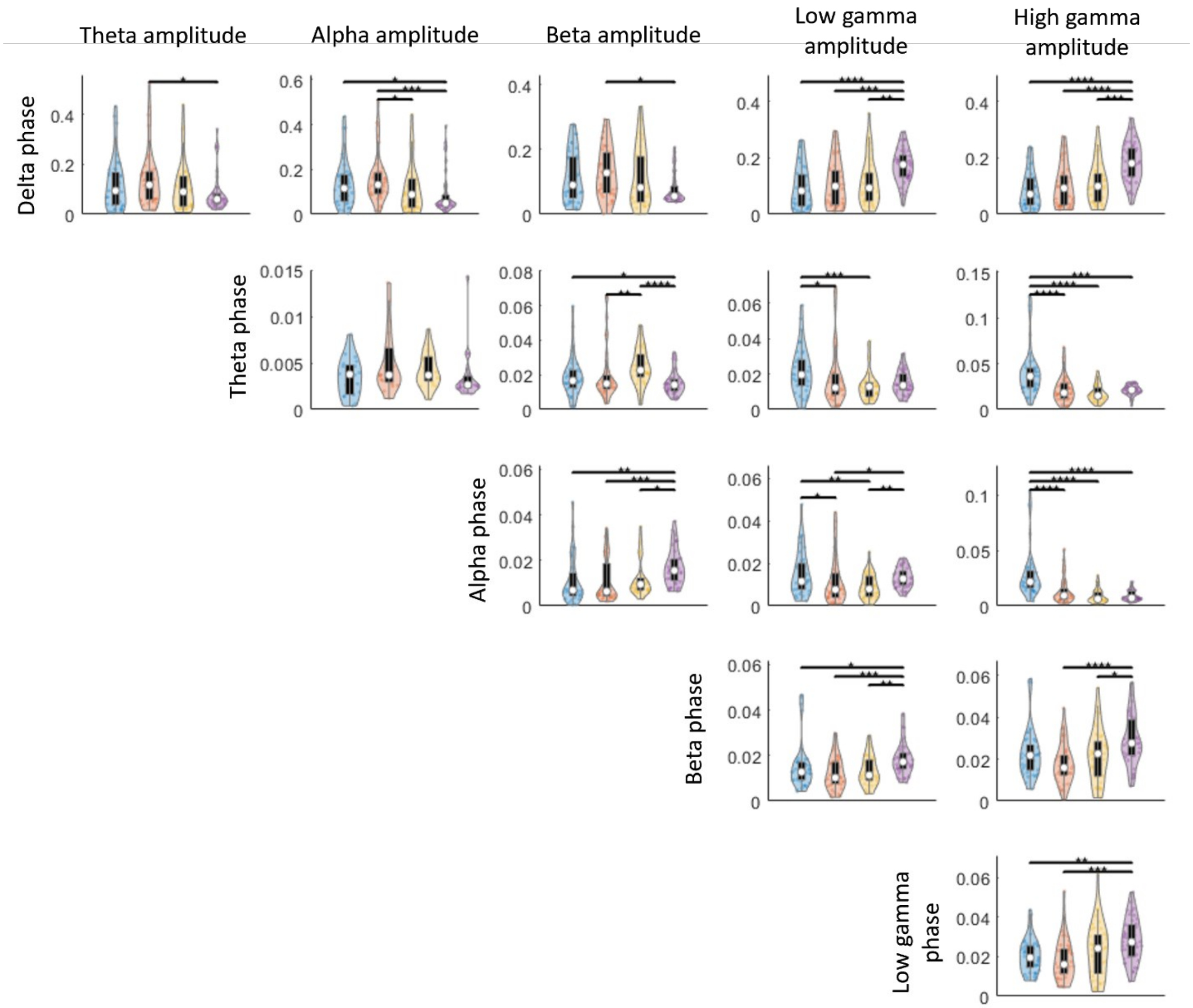

Theta amplitude
Alpha amplitude
Beta amplitude
Low gamma amplitude
High gamma amplitude
Delta phase
Theta phase
Alpha phase
Beta phase
Low gamma phase

Supplementary Figure S7. Changes in spiking patterns for each animal. Average ISI distributions of identified fast spiking and regular spiking neurons during each state (left). Difference of ISI distributions from Move plotted against time (middle) and frequency (right). This figure corresponds to Figures 6A and 6B in the main body of the manuscript.

Monkey K

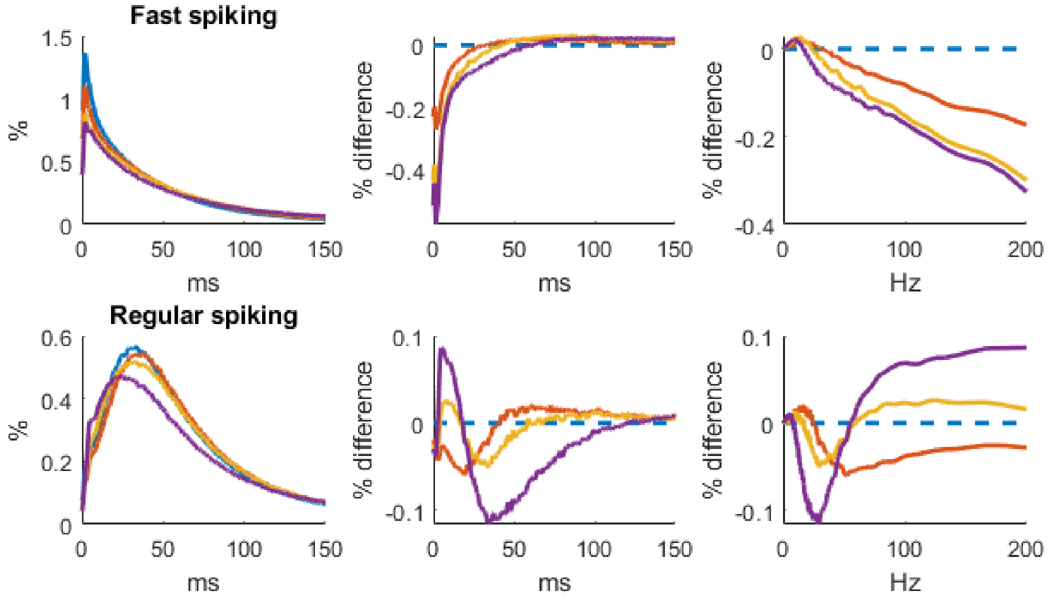

Monkey J

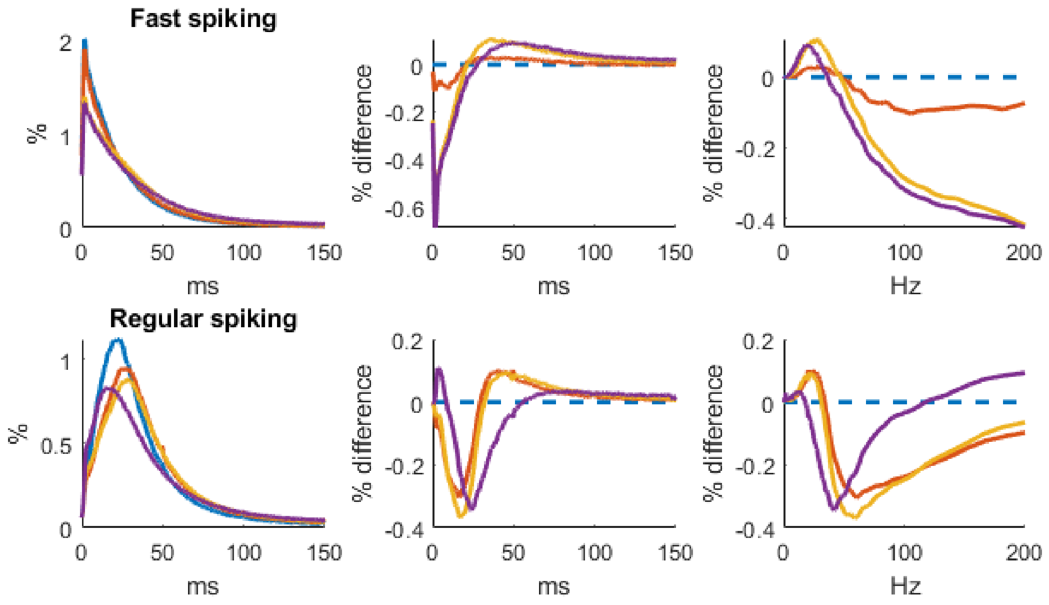

Supplementary Figure S8. Mean vector phase distributions. The distribution of phases of mean vectors for each lower frequency band phase and higher frequency band amplitude pair during each state. The phase of the mean vector shows the phase at which the amplitude of the higher frequency band is the greatest.

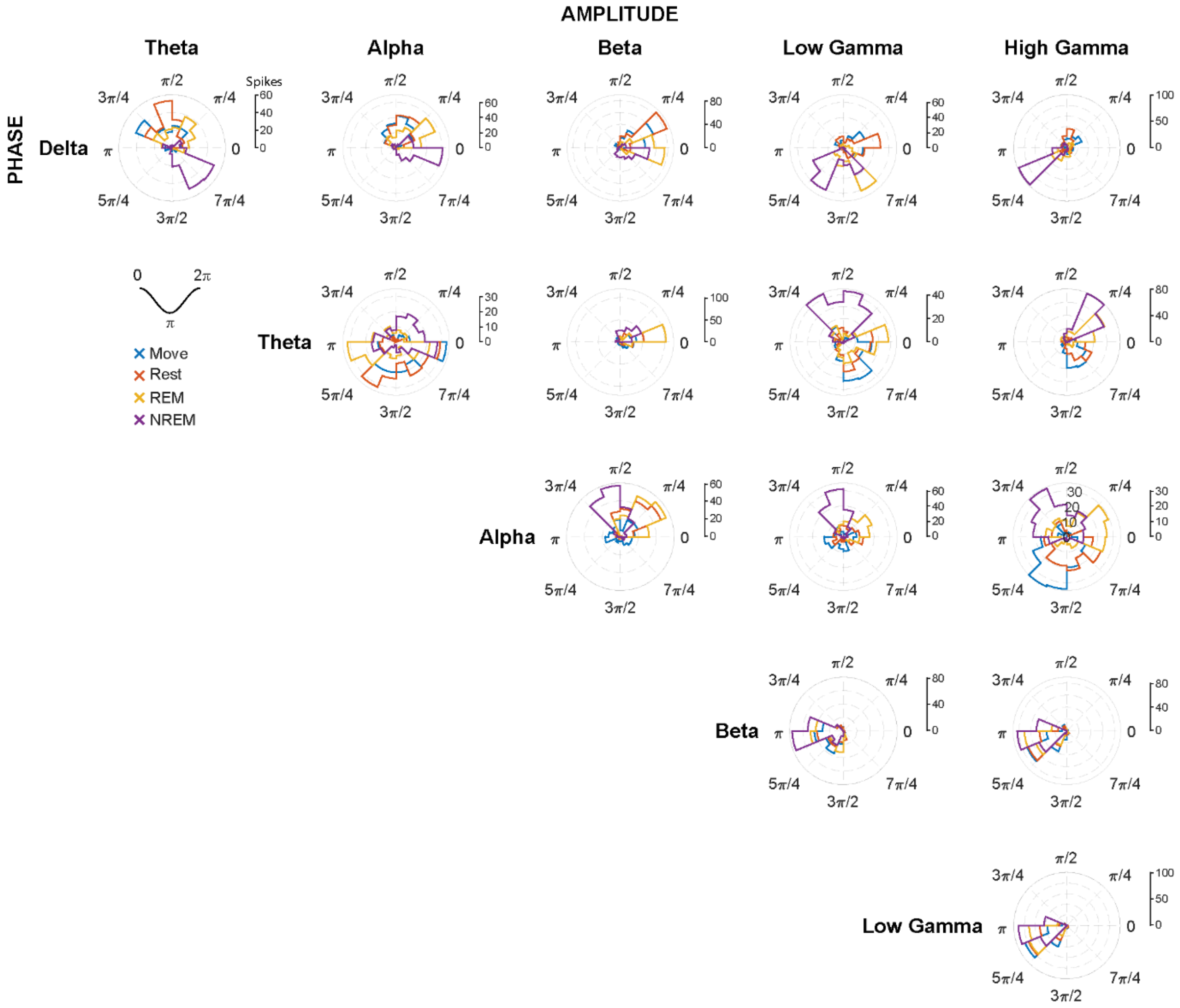

Supplementary Figure S9. Delta phase-power distribution per animal. Average phase-power distribution for delta band LFP in Monkey J (left) and Monkey K (right) normalized to each frequency. The distributions are very similar between the two animals across all four states.

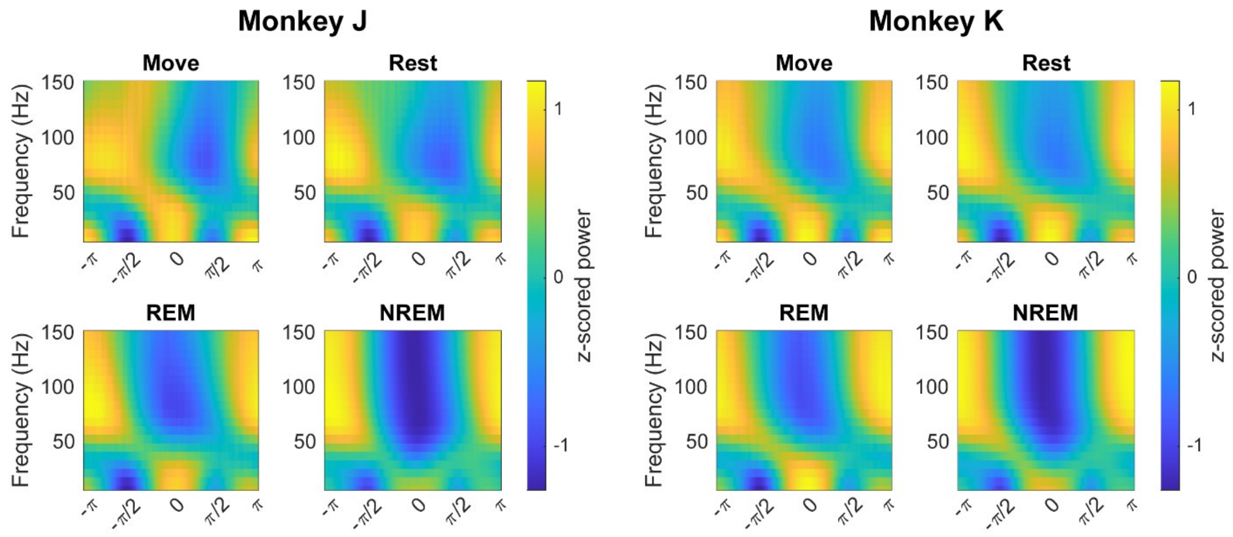

Supplement: Supplementary file 1 [file Data_Sheet_1.pdf]
